# Comparison of model-building strategies for excess hazard regression models in the context of cancer epidemiology

**DOI:** 10.1186/s12874-019-0830-9

**Published:** 2019-11-20

**Authors:** Camille Maringe, Aurélien Belot, Francisco Javier Rubio, Bernard Rachet

**Affiliations:** 1 Cancer Survival Group, Faculty of Epidemiology and Population Health, London School of Hygiene and Tropical Medicine, London, UK; 2 Department of Mathematics, King’s college, London, UK

**Keywords:** Excess hazard models, Interactions, Non-linearity, Non-proportionality, Variable selection

## Abstract

**Background:**

Large and complex population-based cancer data are becoming broadly available, thanks to purposeful linkage between cancer registry data and health electronic records. Aiming at understanding the explanatory power of factors on cancer survival, the modelling and selection of variables need to be understood and exploited properly for improving model-based estimates of cancer survival.

**Method:**

We assess the performances of well-known model selection strategies developed by Royston and Sauerbrei and Wynant and Abrahamowicz that we adapt to the relative survival data setting and to test for interaction terms.

**Results:**

We apply these to all male patients diagnosed with lung cancer in England in 2012 (*N* = 15,688), and followed-up until 31/12/2015. We model the effects of age at diagnosis, tumour stage, deprivation, comorbidity and emergency presentation, as well as interactions between age and all of the above. Given the size of the dataset, all model selection strategies favoured virtually the same model, except for a non-linear effect of age at diagnosis selected by the backward-based selection strategies (versus a linear effect selected otherwise).

**Conclusion:**

The results from extensive simulations evaluating varying model complexity and sample sizes provide guidelines on a model selection strategy in the context of excess hazard modelling.

## Background

Population-based cancer datasets have become richer in recent years. Improved completeness of key variables and additional information from linkages with other datasets (secondary care management data, specialised registry data, treatment data) have both contributed to enhance the quality and utility of data. Furthermore, longstanding datasets make possible the analysis of long-term trends and survival probabilities can be estimated further away from the date of diagnosis.

Analysis of population-based cancer survival has greatly benefitted from this data enrichment. However, when modelling the effect of covariates on survival, special care should be taken when assuming or relaxing assumptions of a linear effect or an effect constant in time (the proportional hazards -PH- assumption). Thus, a modelling strategy is required. Aside from the time-to-event setting, many strategies are developed for variable selection and tests for non-linearity of continuous variables, traditionally based on backward, forward or stepwise algorithms. In the time-to-event field in general, and in population-based cancer survival analyses in particular, less attention has been devoted on the selection of the functional form of predictor variables [1, 2]. Indeed, the effects of variables are commonly assumed linear and constant in time, assumptions likely violated for many predictors of cancer survival, especially with long-term follow-up.

Machine learning algorithms have focussed on variables selection in scenarios where tens or thousands of variables are available [3]. These methods mainly focus on factor analysis and random survival forests [4]. In the context of population-based data, the number of variables remains low or moderate, but the functional forms of their effects (non-linear and/or time-dependent), as well as their possible interactions need to be carefully examined. Model building fits within three different purposes: descriptive, explanatory and predictive [5]. Our aim here is to describe, measure and quantify accurately the effects of relevant (active) variables while excluding spurious effects.

Some authors [6–8] have shown the importance of taking account as well as testing both non-linearity and time-dependency of effects simultaneously, when modelling time-to-event data, in order to get accurate model-based estimates of survival.

We identify two model-building strategies, developed relatively recently, that offer a systematic and comprehensive approach to the selection of predictors’ effects for survival data. One is devised by Sauerbrei and colleagues using fractional polynomials (MFPT) [9] and further adapted for restricted cubic splines (MVRS) [10] and for the inclusion of interactions (MFPI and MFPIgen) [11, 12]. The second one is proposed by Wynant and Abrahamowicz [13], and will be referred to as W&A. These strategies are formulated and tested in the general time-to-event context, in which overall mortality patterns are modelled. Aiming to identify predictors of cancer survival, we focus here on modelling the excess hazard, which is the main quantity of interest in population-based cancer studies [14–16].

Our first aim is to compare and illustrate the use of these model-building strategies (namely MVRS, W&A), in the context of excess hazard regression models. We also propose an extension of those two strategies (called adapted MVRS, aMVRS and adapted W&A, aW&A) for handling interactions between prognostic factors, and compare them to MFPIgen, intended for use with observational data. The performance of these strategies is evaluated in a simulation study mimicking the cancer survival experience of 2000 lung cancer patients diagnosed in 2012 and followed up to the 31/12/2015. We model the effects of explanatory factors on lung cancer survival for the whole cohort of patients diagnosed with lung cancer in 2012. We provide some guidelines over variable and effect selection, based on the simulations.

## Methods

- *The study context: modelling excess mortality*

Our focus is on the excess mortality hazard and the corresponding net survival. The excess mortality hazard is the hazard experienced by cancer patients over and above their background (i.e. expected) mortality hazard due to causes other than the cancer under study. Net survival is derived from the excess hazard and represents the survival experienced by cancer patients under the assumption that they could only die from cancer [17]. Net survival therefore does not depend on the other causes of death, and it is of interest for comparison purposes between countries or periods within a country [18]. In the absence of reliable information on the cause of death, the expected mortality is estimated by the mortality observed in the general population from which patients come from (aka relative survival setting). These life tables are typically defined by age, sex, calendar period, but can also include additional variables such as socio-economic status and ethnicity. Net survival can be estimated non-parametrically [17] or through semiparametric [19] or fully parametric [20–24] excess hazard regression models. Parametric and nonparametric approaches have their own advantages and disadvantages. For the latter, when net survival needs to be estimated in sub-groups, it reduces precision and may lead to unstable estimates. Although there is no assumption relative to the functional forms of effects of variables, these effects cannot be estimated directly. Furthermore, the consistent estimator of net survival proposed by Pohar-Perme and colleagues [17] is unconstrained and thus may show a non-decreasing behaviour in the tails, violating the basic assumptions of survival models. For parametric approaches, the challenges include (a) proper modelling of the baseline excess hazard function, (b) inclusion of potential time-dependent effect of categorical factors, (c) potential non-linear and time-dependent effects of the continuous variables as well as (d) interactions between prognosis factors.

Here, we will use flexible regression models with restricted cubic splines functions for modelling non-linear and time-dependent effects on the log excess hazard scale [23, 25]. The effects of the variables that define the life tables need to be included in the modelling of the excess hazard to produce consistent net survival estimates [17, 20]. Thus, at individual level, the excess mortality hazard *λ*_*E*_(*t*, *x*) is linked to the overall *λ*(*t*, *x*) and expected (population) mortality hazards *λ*_*P*_(*a* + *t*, *y* + *t*, *z*) as follows:

$$ \lambda \left(t,x\right)={\lambda}_E\left(t,x\right)+{\lambda}_P\left(a+t,y+t,z\right), $$

where *z* is a subset of the set of variables *x*, corresponding to the variables defining the life tables, in addition to age *a* + *t* and year *y* + *t* (*a* and *y* being the age at and year of diagnosis, respectively). The population mortality hazard is considered to be known, and we are interested in estimating *λ*_*E*_(*t*, *x*) at time *t* after diagnosis.

In a general form, the excess hazard regression models considered in our work could be written as follows with two prognostic variables *x*_1_, continuous, and *x*_2_, categorical (with *J* categories, *j* = 1, …, *J*):

$$ {\lambda}_E\left(t,x\right)={\lambda}_0(t)\exp \left({\beta}_1(t)\ast f\left({x}_1\right)+\sum \limits_{j=2}^J{\beta}_{2,j}(t)\ast {\mathbbm{I}}_{\left\{{x}_2=j\right\}}\right), $$

where *λ*_0_(*t*) is the baseline excess hazard (defined by using a spline function on the log scale), *f*(*x*_1_) = *α*_1_*x*_1_ if *x*_1_ is modelled with a linear (L) effect, and *f* a spline function of *x*_1_ if *x*_1_ is modelled with a non-linear (NL) effect; *β*_1_(*t*) and *β*_2, *j*_(*t*) are spline functions of *t* if *x*_1_ and *x*_2_ are modelled with time-dependent (TD) effects (the more complicated case), or *β*_1_(*t*) = *β*_1_ and *β*_2, *j*_(*t*) = *β*_2, *j*_, *j* = 1, …, *J* if not (i.e. PH, the simplest case). For the categorical variable *x*_2_, we considered a “joint” parameterisation of its effect: either all *J* − 1 dummy variables are time-dependent, or none. To simplify notation later, we define ${\boldsymbol{\upbeta}}_{\mathbf{2}}(t)\ast g\left({x}_2\right)=\sum \limits_{j=2}^J{\beta}_{2,j}(t)\ast {\mathbbm{I}}_{\left\{{x}_2=j\right\}}$; lastly ${\mathbbm{I}}_{\left\{{x}_2=j\right\}}$ defines an indicator variable (equal to 1 when *x*_2_ = *j*, 0 otherwise).

b. *Model selection strategies*

### The MVRS strategy

*MVRS* is based on an iterative forward selection of variables and increasingly complex functional forms of effects [10]. The model-building proceeds in three steps: (a) the first step focusses on the presence of a variable’s effect, and its possible non-linearity in the case of continuous predictors, while assuming proportionality of hazards for all variables. The iterative process loops through all variables from most to least significant, until no effect is removed or added. (b) In the second step, non-proportionality of hazards is explored by restricting the follow-up time to the time until the median time of observed events on which step (a) is performed and additional effects may be retained. (c) The third step consists of testing the non-proportionality of all effects selected in (a) and (b) in a forward stepwise fashion. The likelihood ratio test is used for evaluating significant effects, with a pre-fixed significance level (usually 5%).

### The W&A strategy

W&A advocate for the use of an iterative backward elimination of non-significant non-linear and time-dependent effects [13]. From the most complex model, including all possible non-linear and time-dependent effects, each non-linear and time-dependent effect is tested in turn using likelihood ratio test, and the effect corresponding to the highest *p*-value (above 5%) is removed. From this new model, we test again each remaining non-linear and time-dependent effect in turn, and repeat those steps until all effects kept are significant. The final model is found when all tests yield *p*-values under 5%.

There are several structural differences in the approaches described above. Firstly, W&A advocates for simultaneous tests of non-linear and time-dependent effects, and the effects are removed one by one, starting from the smallest. By contrast, the MVRS strategy establishes a hierarchy and investigates possible non-linear effects prior to testing time-dependency of the selected effects. The simultaneous tests of effects in W&A may influence subsequent selections of non-linear and/or time-dependent effects. In MVRS, the selection of non-linear effects occurs in the first step, which may well influence the later selection of time-dependent effects, but the selection of time-dependent effects will not affect retention of non-linear effects. Secondly, the initial models considered are different and lead to backward (in the case of W&A) or forward (MVRS) selection of variables.

### Strategies in the relative survival setting

In both strategies, the main life table variables (age, sex, year and deprivation) are forced into the models, as recommended for excess hazard regression modelling [14, 17, 20]. For the non-life table variables linearity and time-dependency and overall effects are tested so the variables could be completely removed from the set of predictors.

### Extensions of the strategies for testing for interactions

The authors of MVRS also consider interactions between variables retained, once the main effects have been selected [11]. MFPI and MFPIgen are defined to consider categorical-by-continuous interactions and continuous-by-continuous interactions respectively, even though (from our understanding) they do not test for non-proportionality of the interaction terms [9].

We propose to adapt the original W&A and MVRS strategies to include tests for the form and presence of interactions in the same fashion that they already test for the functional form and inclusion of each variable.

There are three types of possible interactions: between two continuous variables, between a continuous and a categorical variable, and between two categorical variables. We focus on continuous–by-categorical interaction, and the strategies will need to test whether or not the interaction is needed and if it is time-dependent.

The general form of the excess hazard model is as follows, with *x*_1_ continuous and *x*_2_ categorical (with *J* categories *j* = 1, …, *J*):

$$ {\lambda}_E\left(t,{x}_1,{x}_2\right)={\lambda}_0(t)\exp \left({\beta}_1(t)\ast f\left({x}_1\right)+{\boldsymbol{\upbeta}}_{\mathbf{2}}(t)\ast g\left({x}_2\right)+{\boldsymbol{\upbeta}}_{\mathbf{3}}(t)\ast f\left({x}_1\right)\ast g\left({x}_2\right)\right), $$

with all functions as defined above.

The adapted version of MVRS, aMVRS, tests for each interaction in the three steps presented earlier: (a) joint test of the interaction factors, i.e. test for **β**_**3**_ = 0; (b) In the restricted follow-up time (until the median time of observed events) significance test for **β**_**3**_ = 0; (c) If **β**_**3**_ ≠ 0 in either (a) or (b), test time-dependence of the interaction, i.e. **β**_**3**_(*t*) = **β**_**3**_.

The adapted version of the W&A algorithm, aW&A, tests for each interaction in the same way it tests for the effects of main variables: it first tests for time-dependent effect of the interaction, i.e. **β**_**3**_(*t*) = **β**_**3**_, and then, if a time-fixed effect is favoured, it tests for the main effect of the interaction **β**_**3**_ = 0.

The MFPIgen algorithm only considers interactions in a final step, after selecting the main effects of variables in the usual steps (a)-(c). It tests for **β**_**3**_ = 0. In all algorithms the forms of the interactions, *f* and *g* are defined by the form of the main variables *x*_1_ and *x*_2_ as they are modelled when the interaction is considered.

In the case of interactions with categorical variables, the presence of the interaction could be tested in two different ways: overall (called joint test [26]), or each level of the interaction separately. Here we only test the interactions as one effect, such that all factors relating to one interaction would be removed/included when testing for their inclusion. In the algorithms, the user specifies which interaction terms are worth investigating. Specific significance levels for the tests related to interactions may be chosen as in MVRS. Additional file 1 details how the algorithms are adapted to testing for interactions.

c. *Simulation of biologically plausible lung cancer survival data*

**Box 1:** Summary of the effects simulated

	Age	Stage	Deprivation	Age*Stage
A	L-PH	PH	PH	–
B	L-PH	PH	PH	PH
C	NL-TD	TD	PH	–
D	NL-TD	TD	PH	NL-PH

*L* Linear, *NL* Non-linear, *TD* Time dependent, *PH* Proportional hazards

### Data generation and simulations design

We use the observed survival time and vital status of the full cohort of lung cancer patients (*N* = 17,597), evaluated on the 31st December 2015, to obtain the regression coefficients of an excess hazard regression model. The large sample size enables detection and precise estimation of small effects. These coefficients are used for simulating cancer survival times, as detailed in formulas (A)-(D) below. From this excess hazard regression model, the cancer survival time *Tc* is generated using the inverse transform method [27, 28].

For the data design, we randomly extract 2000 men diagnosed with lung cancer in England in 2012 from the English population-based cancer registry, among those with valid information on stage at diagnosis. We kept the information on their age at diagnosis (continuous variable), their level of deprivation (categorical variable with 5 levels of increasing deprivation measured by the income domain of the Index of Multiple Deprivation [29]), and their stage of cancer at diagnosis (categorical variable with 4 levels of increasing severity based on the Tumour, Nodes, Metastasis classification [30]). The relatively small sample size for population-based data will enable us to test the practical performances of the algorithm in a setting with low censoring rate (15%) but small number of patients (relative to standard population studies). We repeated this for a larger sample of 5000 cancer patients to study the sensitivity of the model selection strategies on the number of events. By default, all results are presented for the samples of 2000 patients, except when clearly mentioned.

We devise four simulation scenarios, representing increasingly complex excess hazard regression models (see Box 1):

(A) Model with linear and proportional effect of age, and proportional effects of stage and deprivation, without interaction

$$ {\lambda}_E\left(t, age, stage, dep\right)={\lambda}_0\left(\mathit{\ln}(t)\right)\ast \exp \left(\alpha \ast age+{\sum}_{i=2:4}{\beta}_i\ast {\mathbbm{I}}_{stage=i}+{\sum}_{j=2:5}{\gamma}_j\ast {\mathbbm{I}}_{dep=j}\right). $$

(B) Model with linear and proportional effect of age, and proportional effect of stage, deprivation and an interaction between age and stage

$$ {\lambda}_E\left(t, age, stage, dep\right)={\lambda}_0\left(\mathit{\ln}(t)\right)\ast \exp \left(\alpha \ast age+{\sum}_{i=2:4}{\beta}_i\ast {\mathbbm{I}}_{stage=i}+{\sum}_{j=2:5}{\gamma}_j\ast {\mathbbm{I}}_{dep=j}+{\sum}_{k=2:4}{\alpha}_k\ast age\ast {\mathbbm{I}}_{stage=k}\right). $$

(C) Model with non-linear and time-dependent effects of age, time-dependent effects of stage, and proportional effects of deprivation, without interaction

$$ {\lambda}_E\left(t, age, stage, dep\right)={\lambda}_0\left(\mathit{\ln}(t)\right)\ast \exp \left(\left(\alpha +{\alpha}^{\ast}\ast \ln (t)\right)\ast f(age)+{\sum}_{i=2:4}\left({\beta}_i+{\beta}_i^{\ast}\ast \mathit{\ln}(t)\right)\ast {\mathbbm{I}}_{stage=i}+{\sum}_{j=2:5}{\gamma}_j\ast {\mathbbm{I}}_{dep=j}\right). $$

(D) Model with non-linear and time-dependent effects of age, time-dependent effects of stage, proportional effects of deprivation and a proportional interaction between age and stage

$$ {\lambda}_E\left(t, age, stage, dep\right)={\lambda}_0\left(\mathit{\ln}(t)\right)\ast \exp \left(\left(\alpha +{\alpha}^{\ast}\ast \ln (t)\right)\ast f(age)+{\sum}_{i=2:4}\left({\beta}_i+{\beta}_i^{\ast}\ast \mathit{\ln}(t)\right)\ast {\mathbbm{I}}_{stage=i}+{\sum}_{j=2:5}{\gamma}_j\ast {\mathbbm{I}}_{dep=j}+{\sum}_{k=2:4}f(age)\ast {\mathbbm{I}}_{stage=k}\right). $$

In the formulas above, associated to scenarios A-D, *f* denotes a restricted cubic splines function with 2 degrees of freedom, i.e. 1 internal knot placed at the median age of the patients’ cohort, *λ*_0_(*ln*(*t*)) is a restricted cubic spline function of time, with up to 3 degrees of freedom, i.e. 2 internal knots placed at the tertiles of the distribution of times to death.

Time to death from other causes *Tp* is generated assuming a piecewise exponential hazard obtained from general population life tables detailed by month of age, sex, calendar month and deprivation level [20]. The censoring time *C* is evaluated on 31/12/2015. The final observed follow-up time for each individual is defined as *T* = *min* (*Tc*, *Tp*, C), with the corresponding vital status indicator *δ* (i.e., *δ* = 0 for censored observations and *δ* = 1 for death).

For each scenario (A-D), we simulate 250 datasets, and we utilise the survsim command in Stata [28] for simulating cancer survival times in the scenarios described above. Close to 90% of patients die in the first four years after diagnosis, classifying lung cancer in the poor-prognosis cancers with low censoring rate.

### Analysis of simulated data

The classical algorithms, W&A and MVRS, are run on scenarios A and C, while the algorithms extended to testing for interactions, aW&A and aMVRS, are run on scenarios B and D. MFPIgen is also tested on scenarios B and D. All excess hazard regression models are fitted using the strcs command in Stata [25], as described in section 2.a.

d. *Indicators used for comparing the model-building strategies*

One additional binary variable not contained in the life tables and absent from the original simulation models is added when testing the model-building strategies. For each scenario, we compare the models selected by each strategy to the original effects used in the simulation with the following indicators.

Firstly, we summarise the proportions of models that select each variable with their non-linear or time-dependent effects for each algorithm. We also study the confounding and self-confounding effects: the impact of mis-specifying one of the components (TD, NL, interactions) of the functional form of a covariable on its other components or on the selection of such components for other variables. We also calculate the proportion of selected models that contain or are exactly the simulated models for each strategy.

Furthermore we provide sensitivity (true positive) and specificity (true negative) values, as defined below, looking at the number of correctly selected effects and the number of correctly unselected effects over the number of active and inactive effects [31]. Both sensitivity and specificity tend to reach 1 for a good estimator:

$$ Se=\frac{\# correctly\ selected\ effects}{\# active\ effects} $$

$$ Sp=\frac{\# correctly\ unselected\ effects}{\# inactive\ effects} $$

Then, for each model building strategy we plot the average of the 250 stage-specific cohort net survival curves and compare them to the true net survival curve. We quantify this comparison by calculating the proportion of the Area Between Curves through time, *pABCtime* [32]*. pABCtime* represents the area between each individual net survival curve (or the average of the 250 net survival curves) and the true generating net survival curve (the reference function). It is expressed as a proportion of the area under the true net survival curve (area under the reference function). A *pABCtime* of 0 % means that the cohort net survival estimates under investigation are in perfect agreement with the true initial observed effect.

For any function *f*, let us assume that the true generating function $\overset{\ast }{f}$and the estimated function $\hat{f}$ cross at time *t*^∗^, *ABCtime* is defined as

$$ ABCtime=\left|{\int}_0^{t^{\ast }}{f}^{\ast }(u) du-{\int}_0^{t^{\ast }}\hat{f}(u) du\right|+\left|{\int}_{t^{\ast}}^T{f}^{\ast }(u) du-{\int}_{t^{\ast}}^T\hat{f}(u) du\right|, $$

and *pABCtime* as

$$ pABCtime=\frac{\left|{\int}_0^{t^{\ast }}{f}^{\ast }(u) du-{\int}_0^{t^{\ast }}\hat{f}(u) du\right|+\left|{\int}_{t^{\ast}}^T{f}^{\ast }(u) du-{\int}_{t^{\ast}}^T\hat{f}(u) du\right|}{\int_0^T{f}^{\ast }(u) du}. $$

*pABCtime* is also calculated for the excess hazard curves estimated for given patients’ factors and for the effects of age, deprivation, and stage comparing the possibly time-dependent estimated HR curves to the originally simulated HR. In such instances, $\hat{f}$ represent the excess hazard, $\hat{f}(u)={\lambda}_E\left(u, age, stage, dep\right)$ or excess hazard ratio, $\hat{f}(u)=\exp \left(\hat{\beta}(u)\right)$.

We also provide bias of effects, at specific time points *t*_*k*_, which are the average bias over all samples (M = 250) between the estimated (possibly time-dependent) effects of age, stage and deprivation and their simulated effects. We specify monthly *t*_*k*_, from diagnosis through to the end of follow-up (4 years):

$$ bias\left(\hat{\beta \left({t}_k\right)}\right)=\frac{1}{M}{\sum}_{k=1}^M\left(\beta \left({t}_k\right)-\hat{\beta \left({t}_k\right)}\right). $$

e. *Application*

We apply the five model-selection strategies (MVRS, W&A, MFPIgen, aMVRS, and aW&A) to our full cohort of 15,688 men diagnosed with non-small cell lung cancer in 2012 in England and followed-up until 31/12/2015. All patients had a minimum potential follow-up of 3 years. Patient’s information on age, deprivation, survival time and vital status is enhanced by information on stage at diagnosis [33] coded using the TNM system (I-IV), emergency route to diagnosis (binary variable) [34], comorbidity status defined after ascertainment of hospital episodes in the 6 months to 6 years prior to diagnosis (binary variable) [35]. The model building strategies test the main effects as well as interactions between age at diagnosis and all other covariates.

All model building strategies yield very similar models (Table 1): no main effect is removed, time-dependent effects of stage, comorbidity and emergency presentation are kept, and when tested, interactions between age and comorbidity is removed by the MVRS algorithm and age and comorbidity and age and emergency presentation by the aW&A and MFPIgen algorithms. Non-linear time-dependent effects of age are retained by the W&A and aW&A algorithms in comparison to linear time dependent effects of age retained in all other model selection algorithms.

**Table 1:** Statistically significant effects of selected prognostic factors identified with each of the five alternative model-building strategies

Variables	aMVRS	MVRS^a^		aW&A	W&A^a^		MFPIgen^b^
Age	L-TD	L-TD		NL-TD	NL-TD		L-TD
Stage	TD	TD		TD	TD		TD
Deprivation	PH	PH		PH	PH		PH
Comorbidity	TD	TD		TD	TD		TD
Emergency diagnosis	TD	TD		TD	TD		TD
Age*Stage	PH	–		PH	–		PH
Age*Deprivation	PH	–		PH	–		PH
Age*Comorbidity		–			–		
Age*Emergency diagnosis	PH	–			–		

*L* Linear, *NL* Non-linear, *TD* Time dependent, *PH* Proportional hazard

^a^Interactive effects not tested

^b^Main effects from MVRS strategy before testing for interaction

**Fig. 1:**
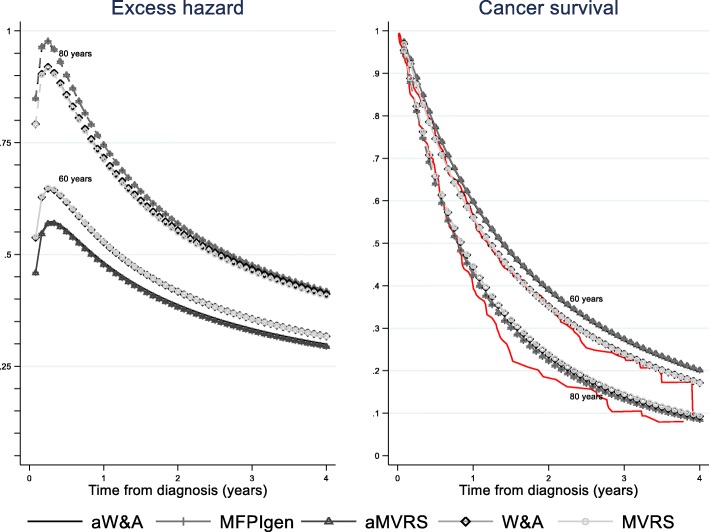
Excess hazard and survival curves estimated for two patients^1^ aged 60 and 80 years at diagnosis: impact of model selection.^1^patient with the following characteristics: stage III, non-emergency presentation, no comorbidity, least deprived. Plain red curves show the non-parametric estimator of net survival for patient aged 50–70 (upper curve) or 75–85 (lower curve) years at diagnosis

Figure 1 illustrates the impact the different selected interactions and linearity/non-linearity of age have on the estimated net survival probabilities for two patients, aged 60 and 80 respectively with the values of other variables set (i.e. stage III, non-emergency presentation, no comorbidity, least deprived). The curves for W&A and MVRS overlap. The selection of interactions in the model impacts the estimated individual excess hazard and cancer survival: there are smaller differences in excess hazard between patients aged 60 and 80 when no interactions are modelled, compared to when interactions are considered. We super-imposed the non-parametric estimator of net survival (red curves) estimated for the 165 patients aged ]50–70[ years (mean age 64) and the 130 patients aged ]75–85[ years at diagnosis (mean age 79), with non-emergency presentation, stage III disease and from the least deprived group of the population. The non-parametric net survival estimates are generally lower than all model-based estimates from 1 year (age 80) and 2.5 years (age 60) after diagnosis. At the start of follow-up, the non-parametric estimates tend to resemble the model-based estimates without interaction terms.

**Fig. 2:**
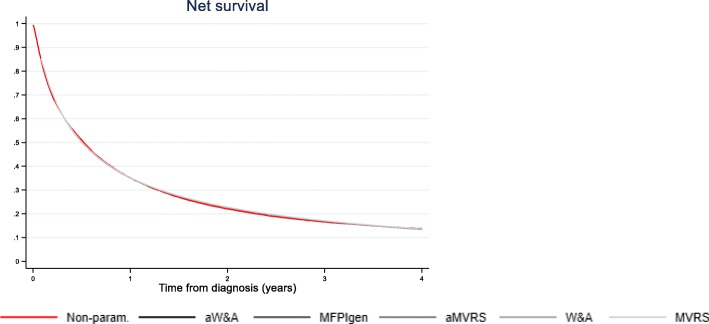
Net survival for the cohort of 15,688 men with lung cancer

These differences at individual level do not however impact the overall cohort estimate of net survival as shown by the hardly distinguishable curves in Fig. 2, similar to the non-parametric estimator of net survival.

## Results

The four simulated scenarios represent increasingly complex but realistic excess hazard models, derived from observed records of lung cancer patients. To assess how realistic these scenarios are, we compare the model-based cohort estimates of net survival (using the model used for each simulated scenario) to the non-parametric Pohar-Perme estimates (Additional file 2) on the original, observed data. All scenarios show reasonable stage-specific cohort net survival estimates. Scenarios A and B under-estimate net survival until 12–24 months for patients diagnosed at stages I-III because of the simple effects modelled. Scenarios C and D include non-proportional effects of the main factors and estimate stage-specific cohort net survival very neatly. The characteristics of the patients used in the simulations are presented in Additional file 3. Patients in stage IV comprise half of the sample. There is a decreasing average age with increasing stage at diagnosis. The distribution of patients by deprivation group is skewed towards more deprived groups, and a third of the patients have the trait of the extra binary variable.

- *Performances of the model-building strategies in selecting variables and their effects*

### Original algorithms – scenarios A and C (no interaction)

**Fig. 3:**
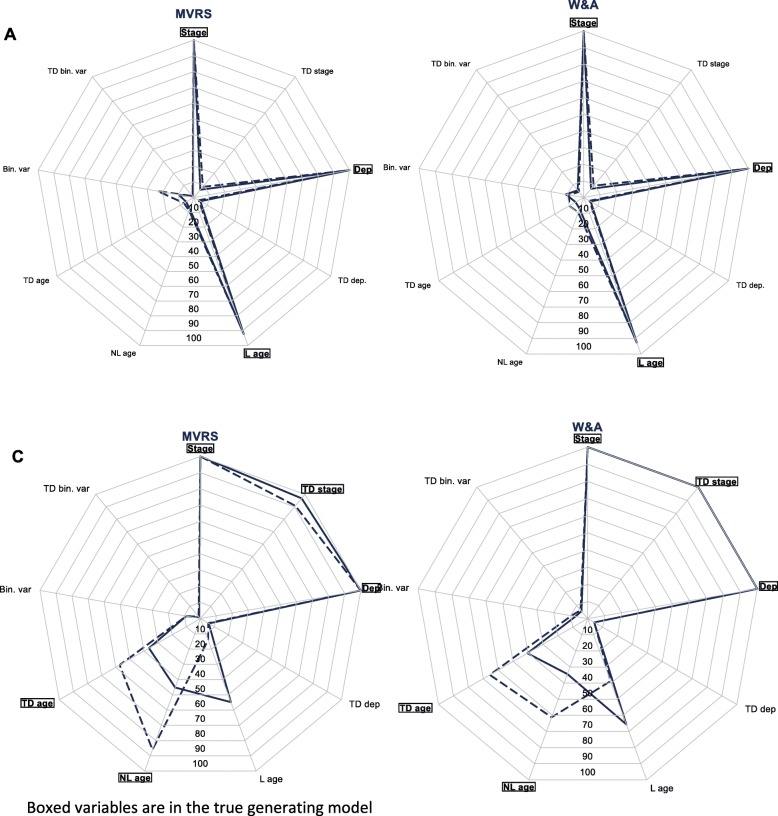
Variables and effects (linear/non-linear, proportional/time-dependent) selected: scenarios A and C, samples of 2000 (plain line) and 5000 (dashed line) patients

In scenario (A), both algorithms led to almost identical selection of effects (Fig. 3, Table 2). The only difference is the higher proportion of time-dependent effects of the extra variable, 5.6% vs. 0.8%, selected with W&A compared to MVRS. In scenario (C), albeit small there are more differences in the effects selected between MVRS and W&A. W&A tends to (rightly) select more models that include time-dependent effects of stage (100% vs. 96.8%) and age (40.4% vs. 36.4%). Non-linear effects of age are more often selected by MVRS (45.2%) than by W&A (34.4%). Overall, the effect of stage is always rightly kept in the final selected models, by all algorithms, and the extra binary variable appears (wrongly) in only 7.2–8.8% of models (Fig. 3, Additional file 4).

**Table 2:** Summary of models and variables selected by each algorithm, on 250 samples of *N* = 2000 and *N* = 5000 patients: scenarios A-D

***N* = 2000**															
	**Overall model**									**Sensitivity**			**Specificity**		
	**Contained**			**Correctly selected**			Almost correctly selected*			**mean**	**min**	**max**	**mean**	**min**	**max**
**A**	**p (%)**	95% CI**		**p (%)**	95% CI**		**p (%)**	95% CI**							
MVRS	100.0	*100*	*100*	69.6	*64.0*	*75.2*				0.97	0.67	1.00	0.89	0.75	0.92
W&A	100.0	*100*	*100*	70.4	*64.8*	*76.0*				0.98	0.67	1.00	0.89	0.67	0.92
**C**															
MVRS	10.8	*7.0*	*14.6*	8.8	*5.3*	*12.3*	82.8	*78.2*	*87.4*	0.74	0.60	0.80	0.88	0.70	0.90
W&A	6.0	*3.1*	*8.9*	5.2	*2.5*	*7.9*	91.6	*88.2*	*95.0*	0.74	0.60	0.80	0.88	0.60	0.90
**B**															
aMVRS	35.6	*29.8*	*41.5*	14.4	*10.1*	*18.7*	53.6	*47.5*	*59.7*	0.80	0.50	1.00	0.85	0.45	0.91
MFPIgen	29.6	*24.0*	*35.2*	14.4	*10.1*	*18.7*	66.8	*61.1*	*72.6*	0.81	0.50	1.00	0.88	0.64	0.91
aW&A	35.2	*29.4*	*41.0*	14.8	*10.5*	*19.1*	45.2	*39.1*	*51.3*	0.79	0.50	1.00	0.84	0.36	0.91
**D**															
aMVRS	2.4	*0.5*	*4.3*	1.6	*0.1*	*3.1*	16.0	*11.5*	*20.5*	0.56	0.50	0.83	0.80	0.33	0.89
MFPIgen	3.2	*1.1*	*5.4*	2.8	*0.8*	*4.8*	36.8	*30.9*	*42.7*	0.59	0.50	0.83	0.85	0.56	0.89
aW&A	4.8	*2.2*	*7.4*	1.6	*0.1*	*3.1*	23.2	*18.1*	*28.4*	0.57	0.50	0.83	0.75	0.22	0.89
***N* = 5000**															
	**Overall mode**l									**Sensitivity**			**Specificity**		
	**Contained**			**Correctly selected**			Almost correctly selected*			**mean**	**min**	**max**	**mean**	**min**	**max**
**A**	**p (%)**	95% CI**		**p (%)**	95% CI**		**p (%)**	95% CI**							
MVRS	100.0	*100*	*100*	56.4	*50.4*	*62.5*				0.97	0.67	1.00	0.88	0.67	0.92
W&A	100.0	*100*	*100*	68.0	*62.3*	*73.7*				0.97	0.67	1.00	0.89	0.67	0.92
**C**															
MVRS	46.0	*39.9*	*52.1*	40.8	*34.8*	*46.8*	78.8	*73.8*	*83.8*	0.78	0.60	0.80	0.88	0.60	0.90
W&A	29.2	*23.7*	*34.8*	26.0	*20.7*	*31.4*	87.6	*83.6*	*91.6*	0.80	0.60	0.80	0.88	0.60	0.90
**B**															
aMVRS	67.9	*62.2*	*73.6*	37.3	*31.5*	*43.3*	55.8	*49.7*	*61.9*	0.80	0.50	1.00	0.87	0.64	0.91
MFPIgen	28.0	*22.5*	*33.5*	14.4	*10.1*	*18.7*	65.6	*59.8*	*71.4*	0.87	0.50	1.00	0.85	0.36	0.91
aW&A	69.2	*63.6*	*74.8*	37.6	*31.7*	*43.5*	55.6	*49.5*	*61.7*	0.86	0.50	1.00	0.82	0.09	0.91
**D**															
aMVRS	34.4	*28.6*	*40.2*	13.6	*9.4*	*17.8*	24.8	*19.5*	*30.1*	0.66	0.33	0.83	0.79	0.33	0.89
MFPIgen	28.0	*22.5*	*33.5*	18.4	*13.7*	*23.1*	35.6	*29.8*	*41.5*	0.69	0.50	0.83	0.84	0.56	0.89
aW&A	22.0	*17.0*	*27.1*	13.6	*9.4*	*17.8*	34.8	*29.0*	*40.6*	0.65	0.50	0.83	0.73	0.00	0.89

* model C: relaxed NL and TD of age; B: relaxed interaction age*stage; D: relaxed NL and TD of age

** formula for the 95% confidence intervals, with *z* = 1.96 and *w* = 250: $\frac{\hat{p}+\frac{z^2}{2w}}{1+\frac{z^2}{w}}\pm \frac{z}{1+\frac{z^2}{w}}\sqrt{\frac{\hat{p}\left(1-\hat{p}\right)}{w}+\frac{z^2}{4{w}^2}}$, using the Wilson approximation [36]

All selected models contain the true simulated model for scenario A but the proportions drop to 69.6% (MVRS) and 70.4% (W&A) of models that are the exact simulated model. Similarly in the slightly more complex scenario (C), 10.8% of models contain, and 8.8% of models are, the true model using MVRS model selection, vs. 6.0 and 5.2% of W&A models, respectively (Table 2). This drop in proportions between scenarios A and C reflects the high proportion of models with a time dependent effect of linear age, in other words the low proportion of models with a time-dependent effect of non-linear age. This is explained by the small sample size and the relatively small magnitude of the non-linear and time-dependent effects of age (Additional file 5). Higher number of lung cancer patients leads to higher proportions of selected models that contain or are exactly the generated model (Table 2) due to higher proportions of models capturing the non-linearity and time-dependency of age (Fig. 3).

Sensitivity and specificity are high for scenario A, and are not impacted by an increasing sample size. They are relatively high for scenario C, with a slight increase in sensitivity (0.74 to 0.78–0.80) with an increasing sample size (Table 2).

### Algorithms adapted to models with interactions –scenarios B and D

**Fig. 4:**
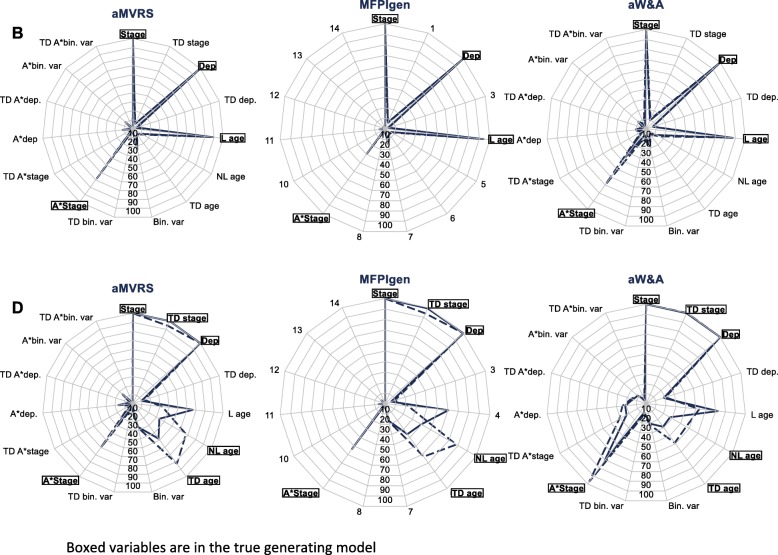
Variables and effects (linear/non-linear, proportional/time-dependent) selected: scenarios B and D, samples of 2000 (plain line) and 5000 (dashed line) patients

The adapted (aMVRS, aW&A) and MFPIgen algorithms correctly keep the main effects in the final models (Fig. 4). 28% of models selected using aW&A identify the non-linearity of age in D, whereas 34–40% of the aMVRS and MFPIgen algorithms retain the non-linearity of age. The aW&A algorithm tends to keep higher proportions of time-dependent effects of deprivation, of the binary variable and of interactions than the other two algorithms. aMVRS and aW&A also lead to 10–21% of interactions wrongly selected. The proportions of the interaction age-stage rightly kept are at or just over 30% for scenario B and up to 71% (aW&A) for scenario D. The MFPIgen algorithm is able to keep in valid interaction in 29.6% (B) and 50.8% (D) of the final models while spurious interactions are rejected in over 94% of final models.

Non-linearity and time-dependency of age in scenario D are retained in just over a quarter of models selected by aW&A, 6–20% less than the proportions of models selected by aMVRS and MFPIgen that contain these characteristics of age*.* Increased sample size to *N* = 5000 is beneficial for raising the detection of the age-stage interaction in B for aMVRS (68.3%) and aW&A (69.2%), and raising detection of non-linearity and time-dependency of age in D for all three algorithms (Fig. 4).

The proportions of models that contain the true generating model lie between 29.6% (MFPIgen) and just over 35% (aMVRS and aW&A) for scenario B, and between 2.4% (aMVRS) and 4.8% (aW&A) for scenario D. For scenario B, those proportions correspond to the proportion of models with an age by stage interaction, and therefore increase with increasing sample size for aMVRS (74.5% for B and 43.5% for D when N = 5000) and aW&A (72.3% for B and 17.4% for D when N = 5000). For scenario D, this is the proportion of models with an interaction between a non-linear effect of age and stage. Only 14.4–14.8% (scenario B) and 1.6–2.8% (scenario D) are the exact simulated models. These proportions increase to 16–36.8% (scenario D) when small effects are not considered, due to the relatively small sample size, or when the sample size is increased to 5000.

Sensitivity and specificity are around and over 0.8 for scenario B and are stable to increased sample size. Sensitivity is just over 0.5, and specificity between 0.75 and 0.85 for scenario D, with slight improvement in sensitivity with increased sample size (Table 2).

### Impact of mis-selection of effects on other effects

In scenario (A) and (C), W&A seems to suffer more from confounding and self-confounding (Additional file 4). For example, when the extra binary variable is selected in (C), the proportion of models with time-dependent effects of deprivation and/or age are hardly changed with MVRS, but they increase with W&A to 16.7% (+ 12.3%) and 55.6% (+ 15.2%) respectively. (Additional file 4).

There are hardly any confounding or self-confounding effects in the MFPIgen algorithm. Mis-specification of time-dependent effects only has minimal confounding impact on the other effects selected using the aMVRS algorithm. This is due to the two-step structure of the algorithm (Additional file 4).

In the aW&A algorithm, selection of complex forms (e.g. time-dependent effect of a variable) results on the selection of more complex effects of some other factors or additional selection of interaction terms (Additional file 4).

(b) *Accuracy of the non-linear and time-dependent effects estimated*

### Original algorithms – scenarios A and C (no interaction)

**Fig. 5:**
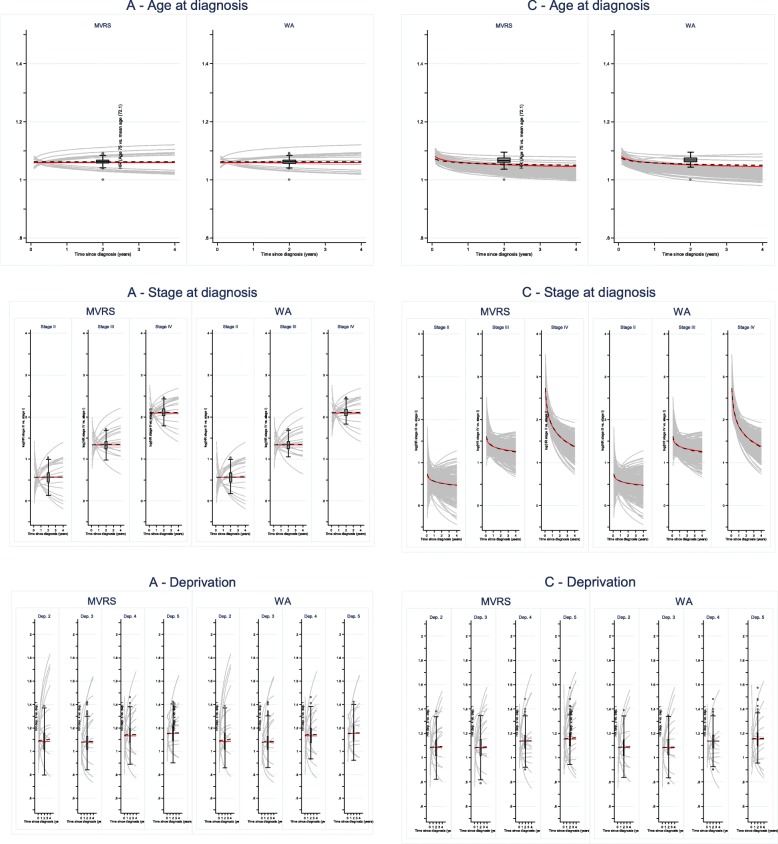
Generating (red line), estimated (grey lines - time varying, box-plot - time fixed) and averaged (black dashed line) hazard ratio for age, stage, and deprivation: scenario A and C

Figure 5 presents the effects estimated by the models selected following the MVRS or W&A algorithms together with their averaged effects (black line) compared to the true generating effect (red line). All sample sizes are *N* = 2000 patients.

Although there are varied sizes of effect estimated as shown by the width of the boxes (effects estimated as fixed in time) and the varied shapes of the individual effects, grey curves (time-dependent effects estimated), the average effects generally agree with the generating effects for all strategies, and lead to comparable estimated effects for MVRS and W&A. For both strategies, the effects of age are well captured for scenario (A) and (C): *pABCtime* values are 0.3% (A), 0.3% (C, MVRS) and 0.2% (C, W&A), Table 3.

**Table 3:** *pABCtime* between the mean of the individual effects or cohort net survival estimated using the selected models and the true generating effects/cohort net survival, by scenario (A-D), and model selection strategy

**Cohort net survival**	*Stage*	A	B	C	D	**HR age**	*Stage*	A	B	C	D
MVRS / aMVRS	I	1.67%	1.62%	0.79%	1.09%	MVRS / aMVRS	I	0.34%	2.43%	0.35%	4.40%
	II	0.94%	1.60%	0.99%	1.10%		II		3.56%		5.86%
	III	0.56%	0.53%	0.16%	1.37%		III		0.01%		0.73%
	IV	0.36%	0.36%	0.11%	0.31%		IV		0.13%		2.15%
W&A / aW&A	I	0.05%	1.63%	0.04%	0.89%	W&A / aW&A	I	0.33%	2.35%	0.23%	2.88%
	II	0.20%	1.60%	0.01%	1.00%		II		3.45%		4.13%
	III	0.06%	0.54%	0.94%	1.18%		III		0.02%		0.61%
	IV	0.13%	0.37%	0.68%	0.08%		IV		0.12%		1.26%
MFPIgen	I		0.09%		0.08%	MFPIgen	I		2.55%		2.73%
	II		0.13%		1.11%		II		3.76%		3.92%
	III		1.18%		0.99%		III		0.01%		0.63%
	IV		0.21%		0.71%		IV		0.06%		1.86%
**HR stage**	*Stage*	A	B	C	D	**HR deprivation**	*Deprivation*	A	B	C	D
MVRS / aMVRS	I					MVRS / aMVRS	2	1.20%	0.03%	0.34%	1.38%
	II	2.25%	3.30%	2.44%	1.01%		3	0.23%	0.03%	0.39%	0.75%
	III	1.71%	1.75%	1.20%	2.96%		4	0.93%	0.40%	0.21%	1.24%
	IV	2.49%	1.93%	2.15%	5.49%		5	0.13%	0.26%	0.53%	1.36%
W&A / aW&A	I					W&A / aW&A	2	1.20%	0.02%	0.26%	1.20%
	II	2.35%	3.36%	2.32%	1.71%		3	0.27%	0.01%	0.35%	0.57%
	III	1.66%	1.86%	0.85%	3.42%		4	0.95%	0.23%	0.12%	0.84%
	IV	2.52%	2.08%	1.80%	5.24%		5	0.13%	0.50%	0.28%	1.20%
MFPIgen	I					MFPIgen	2		0.03%		1.81%
	II		3.25%		1.39%		3		0.02%		1.02%
	III		1.67%		3.36%		4		0.44%		1.11%
	IV		1.83%		5.37%		5		0.23%		1.38%
**HR comorbidity**		A	B	C	D						
MVRS / aMVRS		0.31%	0.22%	0.08%	0.18%						
W&A / aW&A		0.59%	0.34%	0.12%	0.12%						
MFPIgen			0.36%		0.17%						

The mixture of time-fixed and time-dependent effects of stage estimated in the selected models for scenario (A) leads to a very good estimation of the average effect compared to the generated effect for both strategies. Note the graphs present log hazard ratios for better illustrating the differences, but *pABCtime* values are calculated on the areas between the hazard ratio curves. *pABCtime* values for the hazard ratios are very similar between algorithms, highest for stage IV (2.5%), intermediate for stage II (2.2–2.4%) and lowest for stage III (1.7%). In scenario (C) all estimated effects are time-dependent, and most shapes agree with the original effect. *pABCtime* values are slightly lower for the W&A algorithm compared to MVRS: 2.3% vs. 2.4% at stage II vs. I, 0.9% vs. 1.2% at stage III vs. I, and 1.8% vs. 2.1% at stage IV vs. I.

The effects of deprivation are well estimated by all models selected by all algorithms: *pABCtime* is below 1.2% for all deprivation categories, and in both scenarios A and C.

More complex effects of the extra binary variables are captured by W&A, in both (A) and (C) leading to slightly higher *pABCtime* values: 0.6% vs. 0.3% (A) and 0.12% vs. 0.08% (C).

### Algorithms adapted to models with interactions – scenarios B and D

**Fig. 6:**
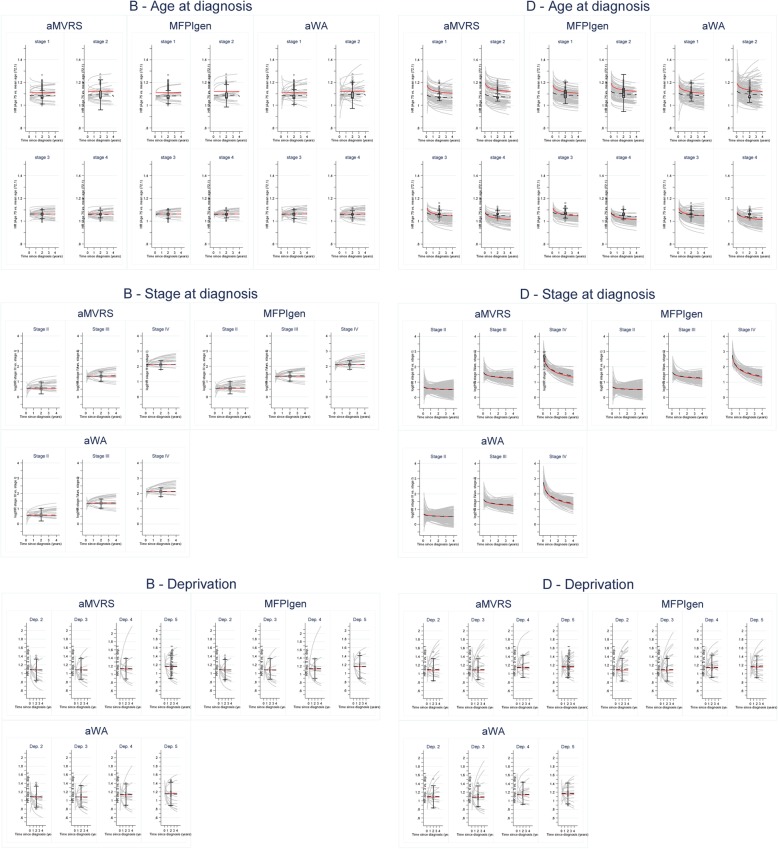
Generating (red line), estimated (grey lines if estimated as time varying, or in the box-plot if estimated as time fixed) and averaged (black dashed line) hazard ratio for age, stage, and deprivation: scenario B and D

Figure 6 displays the effects estimated by the selected models (250 grey curves) following the aMVRS, MFPIgen and aW&A algorithms together with their averaged effects (black line) compared to the true generating effect (red line). The effects of age are now split by stage at diagnosis, since an interaction age-stage is simulated.

For all selected models, the average HRs for age seem to generally underestimate the simulated effects for stages I-II, in scenario B and D. These are reflected by larger stage-specific *pABCtime* values for age: 2.4–5.9% (stages I-II) versus 0.01%-2.2 (stages III-IV, Table 3). The time-dependency of age, simulated in scenario D, is not very strong, hence the many models that selected a time-fixed effect for age. Graphs of the non-linear effects of age at given times after diagnosis are presented in Additional file 5.

The effects of stage, deprivation (Fig. 6) and the additional binary variable (Additional file 6) are well reproduced by the average effects obtained from the selected models. The *pABCtime* values can hardly distinguish between the performance of the model-selection algorithms (Table 3). The complexity of models selected by the aW&A algorithm does not impact the overall measures of effects and their adequacy to describe the true generating effects. Indeed, none of the modelled time-dependent effects are strong, but the results presented here shed some light in terms of the sensitivity of the different model selection tools.

(c) *Estimation of the cohort net survival*

For all model-building strategies, the estimated stage-specific cohort net survival curves lie around the original estimated cohort net survival curves, for all subgroups defined by stage at diagnosis for scenarios A-D (Fig. 1). All *pABCtime* values are below 1.7% (Table 3).

The outcome of choice – net survival – is well reproduced by models selected by each strategy and provides reassurance that the experience of cancer survival for the cohort is well captured by the models. *pABCtime* values calculated using the non-parametric estimator of net survival provides 0.3–8% higher values than for the model-based survival curves (Additional file 7).

The bias reflects the varying amount of mis-specification for each of the three algorithms. For example, higher proportions of time-dependent effect of the binary variables using W&A and aW&A lead to higher standardised bias for that variable and that algorithm (Additional file 8). The minimum in the time-varying bias is reached at around 6 months after diagnosis for all effects, when most time-dependent effects cross the true effect. At that point, the value reached reflects the amount of bias due to the estimated fixed effects.

## Discussion

Motivated by the growing access to data on explanatory factors of cancer survival, we compared the practical use of several model selection strategies. We adapted well-recognised algorithms to the context of excess hazard models, including extensions to deal with 2-way interactions. Simulations, based on observed realistic scenarios, showed the ability of all strategies to yield proper estimation of the cohort net survival curve despite varying forms of the retained and estimated effects.

Several aspects of model selection deserve further discussion. Additionally, we aim to provide some guidelines for variable selection in the context of cancer survival epidemiology.

### Subject matter knowledge

A breadth of modelling strategies exists, but very few strategies have been compared as highlighted by STRATOS Topic Group 2 [37]. We aimed here to look at the impact that model selection strategies may have on inference based on the final selected model. Subject matter knowledge is needed all through model building, such as in decisions relative to the selection of the variables that will be tested, and the allowed forms of these variables [38], as well as how strict we are on keeping/dropping a variable or functional form. In observational studies, we acknowledge it is almost impossible to state all aspects of a model ahead of data exploration, and model selection remains necessary. In our comparison, we concentrate on the model-building algorithms per se and assume both benefited from a similar amount of subject matter knowledge.

### Time-dependent effects

A time-dependent effect is modelled if the effect of a variable, measured at diagnosis, varies with time since diagnosis, i.e. that effect is not constant with follow-up time. In the context of cancer survival, most factors such as stage at diagnosis, deprivation, emergency presentation [39] tend to have strong effects in the months that follow the diagnosis, and these effects are likely to reduce or disappear as time passes [39]. When testing time-dependency of different factors, a long enough follow-up, as well as enough information are required to detect time-dependency.

### Non-linear effects

Additionally, in order to properly assess non-linearity of the effect of a specific variable, such as age, there needs to be enough information on that variable about its own effect on the time to event: e.g. patients’ age need to cover a reasonable range of all possible ages, rather than be grouped in a small part of the age distribution.

### Censoring and lethality of cancer (number of events)

Lung cancer data contain relatively high proportions of events (80% 4 years after diagnosis) compared to other cancers that do not experience such high lethality. Model building strategies and variable selections are highly sensitive both to the number of events and levels of censoring. This is due to the rapidly increasing complexity of the models tested, especially when the backward-based W&A and aW&A are run. For example, in the context of lung cancer, there was non-convergence of the Stata algorithms in around 10% of the samples. Changing the starting values or running initial univariate selections did not help in reaching convergence.

It has recently been shown that 40–50 events per variable are necessary to ensure accurate estimation of coefficients [40] in the competing risk setting. In the most complex models (fitted on lung cancer) which include all interactions and time-dependent effects, i.e. 48 parameters, there was an average of 36 events per parameters in a sample of 2000 patients. When these model-building strategies were run on cancers with lower lethality, such as laryngeal cancer, with 60% censoring at 5 years, the algorithms did not converge for a larger proportion of samples, up to 20% (results not shown). In addition, after convergence, some estimated hazard ratios were unbelievably large: there was an average of only 16 events per parameters (*N* = 2000 patients) for the most complex models fitted on laryngeal cancer data.

In the relative survival data setting, a competing risk framework, competing deaths (i.e. from other causes, provided by general population life tables) are subtracted from observed events (death from any cause). This reduces further the power for detecting and retaining effects. This is not so problematic when studying lung cancer as 95% of deaths are due to lung cancer [39], i.e. 1675 lung cancer deaths among the 1765 deaths in the 2000 data samples, leading to 34 events per parameter. Less lethal cancers will see the actual numbers of cancer-related deaths be a smaller proportion of all deaths, leading to smaller number of events per variable.

Prior to running any model building strategies, we recommend that the censoring rate and the number of events are carefully examined in relation to the complexity of the models fitted. Further clinical considerations and background knowledge are helpful prior to variable selection to ensure significance tests are used with sparsity.

### Sample size, model complexity

The W&A strategies tend to favour time-dependent effects and interactions, leading to complex models. This is due to the backward selection of effects. Model misspecification of some variables leads to self-confounding and confounding, which would provide wrong inference on the effects of some variables. On the other hand, the MVRS strategy leads to simpler models with additional variables wrongly selected in about 5% of models overall. However, in three out of four scenarios (B, C and D), all model selection strategies select models containing the true models in a relatively poor proportion (always below 15%). This is largely due to the size of the effects that the algorithms were trying to capture and the number of patients included in the analyses, 2000. Indeed, some effects such as non-linearity or non-proportionality of age could not be retrieved in the final selected models, due to lack of power. Releasing one or several of these small effects translates in larger proportions of models that nearly contain the generating models. More importantly, increasing the sample size to 5000 patients leads to improved detection power and higher selection proportions of the true generating model.

The adapted MVRS and W&A algorithms testing for interactions show similar properties as the original algorithms for the selection of linear/non-linear and time-dependent main effects. They show equivalent results to the MFPIgen strategy for the selection of interaction terms.

Investigating the effect of many variables of known prognostic value in a large population-based cohort of lung cancer patients, all model-building strategies lead to similar selection of effects. As expected W&A and aW&A only differed from R&S and aR&S in the shape of the effect of age, which has virtually no impact on cohort-wide net survival estimates.

Although the model-building strategies may not tend to select the same final models, and the proportion of models that do select the true generating model vary with the sample size, the number of events and the size of the effects, there is no impact on the estimation of cohort net survival, by stage at diagnosis. Estimation of cohort net survival can best be done non-parametrically as there is no assumption on the form of the association between the exposure variables and survival time. We show that on average the model-based estimates are equivalent to the non-parametric estimates of net survival. When non-parametric estimates of cohort survival can be produced, it is good practice to use them to validate model-based estimates.

The variables whose effects are tested in the models, are only mildly correlated with a coefficient of correlation below 0.2. Another challenge in modelling non-linear effects of a variable is the potential collinearity of some spline basis (such as cubic splines). A possible solution for this, adopted here, consists of orthogonalizing the splines basis. However, high correlation between two variables may have a negative effect on the model selection strategies studied here as they are based on stepwise methods and are thus dependent on the order of testing.

### Epidemiological aim of models

The ultimate aim of building exploratory models in our context is to describe variables effects on the survival experience of a cohort of cancer patients. In the simulations, the large variety of models selected by the different model-building strategies leads to varying estimations of main effects and varying levels of individual excess hazard and net survival estimates, which has implications in terms of epidemiological interpretation. Nonetheless all generated effects are well captured by the variable selection strategies, whatever their complexity. This is verified graphically and looking at the area between each estimated effect and the generated effect.

Forward-based model building strategies tend to favour simpler models, which may be a useful feature in contexts with less information (e.g. low EVP, or high censoring, or relatively small sample sizes) in order to avoid inclusion of spurious effects. Conversely, backward-based strategies tend favour more complex models, which may be useful to detect small effects in cases with larger samples and low censoring. Nonetheless, the comparison of the final models selected with different strategies may be useful in order to assess any differences on the corresponding net survival curves, and to identify potential reasons for these differences (if any) based on our previous discussion.

The strategies presented here are based on likelihood ratio tests performed in a hierarchical order. Thus, they rely on significance testing and, consequently, are prone to multiple testing as well as Type I and Type II errors. Nonetheless, all strategies let the user decide what significance level should be used for the selection of effect. We use here the conventional 5%, and test for the impact of keeping the main effects in. One could consider choosing more conservative thresholds [41] and evaluating the impact of varying thresholds on the models selected.

Model building strategy is in line with the ‘data modelling culture’ and is based on the idea that *a true* model generating the data does exist [42]. Although not all important variables may be available, or the true model is likely to not be among the considered models, the aim is to get as close as possible to this true model by including the relevant variables and by flexibly modelling the effect of the available ones. Shrinkage techniques (LASSO [43], Ridge, Elastic Nets [44]) could be considered, but these methods are not yet available in our relative survival context. Still in the machine learning field, methodological developments are of great interest. For example, model averaging [45] and more generally ensemble learning techniques [46] are possible avenues though interpretability of the results can be challenging, hence more appropriate outside of the descriptive modelling field.

Model selection approaches based on Information Criteria [45] (e.g. AIC and BIC) or cross-validation of the selected models, instead of likelihood ratio testing, could prove useful for selecting the proper functional forms of effects. In the context of prediction, one tends to select and use a simple model in order not to over fit the training data [47]. Following work on the topic of predictions would involve additional statistical measures for assessing predictive accuracy of the selected model for a given strategy. Measures such as discrimination and calibration would then be useful [48, 49]. However, in this work, which was mainly exploratory rather than predictive, all strategies lead to similar model-based estimates of net survival.

Large datasets and information on many factors are motivations for using complex excess hazard models. Model selection methods are essential to make sure all models are considered in a systematic fashion. Nonetheless, several aspects of the data (such as sample size, censoring, NL and TD effects) and the models (such as complexity, assumptions) deserve full consideration ahead of model selection.

## Additional file

**Additional file 1.** Adaptation of the W&A and MVRS for testing for interactions.

**Additional file 2.** Comparison of model-based estimates of survival and the non-parametric Pohar Perme estimator of survival.

**Additional file 3.** Descriptive statistics for the sample of patients used in data simulations.

**Additional file 4.** Self-confounding and confounding effect due to mis-selection of variable effects (scenario A and C).

**Additional file 5.** Effects of age, generating (red), estimated (grey), averaged (black) for all models selected by each algorithm, scenario A-D.

**Additional file 6.** Effect of the extra binary variable in the models selected by each algorithm, scenario A-D.

**Additional file 7.** Original (red line), estimated (grey lines - time varying, box-plot - time fixed) and averaged (black line) cohort net survival. Scenario A-D.

**Additional file 8.** Integrated absolute estimated bias for the effects of age, stage, deprivation and the extra binary variable, by scenario (A-D) and for each model selection algorithm.

## Data Availability

The data that support the findings of this study – both in simulations and application – are available from Public Health England but restrictions apply to the availability of these data, which were used under the necessary statutory and ethical approvals for the current study, and so are not publicly available.

## Abbreviations

AIC: Akaike Information Criteria
BIC: Bayesian Information Criteria
LASSO: Least Absolute Shrinkage and Selection Operator
MFPI: Multivariable fractional polynomial (interaction)
MFPIgen: Multivariable fractional polynomial (general interaction)
MFPT: Multivariable fractional polynomial (time)
MVRS: Multivariable regression splines
NL: Non linear
pABCtime: Proportion of the Area Between Curves through time
PH: Proportional Hazards
TD: Time dependent
W&A: Wynant and Abrahamowicz
