# Supplementary material for: Comparison of model-building strategies for excess hazard regression models in the context of cancer epidemiology
Source: BMC Med Res Methodol. 2019 Nov 20;19:210. doi: 10.1186/s12874-019-0830-9 (PMC6869178; doi:10.1186/s12874-019-0830-9)
Supplement: Supplementary file 1 — Additional file 1. Adaptation of the W&A and MVRS for testing for interactions. [file 12874_2019_830_MOESM1_ESM.docx]

**Additional file 1**

Adaptation of the W&A and MVRS for testing for interactions

|  | ***adapted* Wynant and Abrahamowicz aW&A** | ***adapted* MVRS aMVRS** |
| --- | --- | --- |
|  |  |  |
| **Initial setting** | * Nominal p-values for selecting: - Main effects of LT variables: 1 - Main effects of other variables, and complexity of effects (non-linearity for continuous factors and non-proportionality for all factors): α | * Nominal p-values for selecting: - Main effects: α1 (α1=1 forces variable into the model) - Complexity of effects, non-linearity for continuous factors and non-proportionality for all factors: α2 (α2=1 most complex permitted function kept) - Interactions, α1'. * Maximum number of cycles: c_max |
| **Initial model** |  |  |
|  | Most complex model M1 fitted: - all main effects with non-proportionality and non-linearity - all possible interactions, with non-linearity No convergence, remove: - non-proportional interaction term, one by one successively - non-proportional main effect, one by one successively - each interaction, successively | Full linear proportional model is fitted. NB: The ‘visiting order’ of the predictors is determined according to the p-value for omitting each predictor from the model. The most significant predictor is visited first and the least last. We assume that variables x1,…,xn have been arranged in this order, retained in all cycles of the procedure. |
| **Cycle counter c = 0** |  |  |
|  |  |  |
| **For each x, continuous main effects** | |  |
|  | * x is linear proportional and not involved in interactions: - test for main effect of x: M1 *vs*. a model that excludes x. Keep p-value. * x is non-linear: - test for linearity: M1 *vs*. M1 including a linear effect of x (in both main effect and interaction(s)). Keep p-value. *x is non-proportional: - test for proportionality: M1 *vs*. M1 excluding non-proportional x. Keep p-value. | * Test non-linear x against excluding x if x is not involved in interactions, at the α1 level. - If not significant, x is dropped. - Else, test non-linear *vs*. linear x, at the α2 level.  If not significant, the final effect of x is linear, else, x is non-linear. |
| **For each x, categorical main effect** | |  |
|  | * x is proportional and not involved in interactions: - test for main effect of x: M1 *vs*. a model that excludes x. Keep p-value. * x is non-proportional: - test for proportionality: M1 *vs*. M1 excluding non-proportional x. Keep p-value. | * If x is not invovled in interactions, joint significance of the dummy variables tested at the α1 level. - If significant, it is included. - Else, it is dropped. |
| **For each x, interaction** | |  |
|  | * x is non-proportional: - test for proportionality: M1 *vs*. M1 excluding non-proportional x. Keep p-value. * overall: - test for the main effect of x: M1 *vs*. a model that excludes x. Keep p-value. | * Test model including x against a model excluding x, at the α1' level. - If not significant, x is dropped. Else, x is included as is. |
|  |  |  |
|  | * Compare all p-values obtained in that cycle. * Remove the effect that leads to the highest p-value, if that p-value is over α | * Changes to x apply until x is reconsidered in the next cycle |
| **Let c = c+1. The *c*th cycle is complete** | |  |
|  | If no effect was removed between cycle c and cycle c+1, the final selected model is found. | * If c>c_max, stop and report that the algorithm failed to converge in c_max cycles. * Check whether included variables and non-linear transformations have changed from cycle c and cycle c+1.  - If so, start a new cycle.  - If not report current model as M0. End. |
|  |  | **Selection of time varying effects:**  * Run the above algorithm on [0;τ] where τ is the time at which half of the events occur. * Add to model M0 the variables and forms of variables that were selected to form model M1. * For each variable in M1:  - test non-proportionality using a forward stepwise approach at the α3 level If significant, keep non-proportional effect of x. |
