# Supplementary material for: Comparison of model-building strategies for excess hazard regression models in the context of cancer epidemiology
Source: BMC Med Res Methodol. 2019 Nov 20;19:210. doi: 10.1186/s12874-019-0830-9 (PMC6869178; doi:10.1186/s12874-019-0830-9)
Supplement: Supplementary file 2 — Additional file 2. Comparison of model-based estimates of survival and the non-parametric Pohar Perme estimator of survival. [file 12874_2019_830_MOESM2_ESM.docx]

**Additional file 2**

Comparison of model-based estimates of survival and the non-parametric Pohar Perme estimator of survival
