# Supplementary material for: Comparison of model-building strategies for excess hazard regression models in the context of cancer epidemiology
Source: BMC Med Res Methodol. 2019 Nov 20;19:210. doi: 10.1186/s12874-019-0830-9 (PMC6869178; doi:10.1186/s12874-019-0830-9)
Supplement: Supplementary file 3 — Additional file 3. Descriptive statistics for the sample of patients used in data simulations. [file 12874_2019_830_MOESM3_ESM.docx]

**Additional file 3**

Descriptive statistics for the sample of patients used in data simulations

|  |  |  | **Stage I** | |  | **Stage II** | |  | **Stage III** | |  | **Stage IV** | |
| --- | --- | --- | --- | --- | --- | --- | --- | --- | --- | --- | --- | --- | --- |
| **N (%)** |  |  | 255 | (12.6) |  | 164 | (8.1) |  | 505 | (25.0) |  | 1098 | (54.3) |
|  |  |  |  |  |  |  |  |  |  |  |  |  |  |
| **Age at diagnosis** | |  |  |  |  |  |  |  |  |  |  |  |  |
|  | Mean age |  | 73.5 | |  | 73.7 | |  | 71.9 | |  | 72.0 | |
|  | Standard deviation | | 9.4 | |  | 9.3 | |  | 10.3 | |  | 10.4 | |
|  | Min age |  | 29.6 | |  | 45.1 | |  | 42.9 | |  | 24.2 | |
|  | Max age |  | 94.4 | |  | 93.9 | |  | 94.0 | |  | 97.9 | |
|  |  |  |  |  |  |  |  |  |  |  |  |  |  |
|  | prop. of patients aged over 85 years at diagnosis |  | 9.8 | |  | 12.2 | |  | 10.5 | |  | 9.4 | |
|  |  |  |  |  |  |  |  |  |  |  |  |  |  |
| **Deprivation (%)** | |  |  |  |  |  |  |  |  |  |  |  |  |
|  | Least deprived |  | 17.3 | |  | 14.0 | |  | 12.7 | |  | 14.8 | |
|  | 2 |  | 15.3 | |  | 15.9 | |  | 18.0 | |  | 15.9 | |
|  | 3 |  | 20.4 | |  | 23.2 | |  | 19.0 | |  | 19.9 | |
|  | 4 |  | 19.6 | |  | 25.0 | |  | 24.2 | |  | 22.4 | |
|  | Most deprived |  | 27.5 | |  | 22.0 | |  | 26.1 | |  | 27.0 | |
|  |  |  |  |  |  |  |  |  |  |  |  |  |  |
| **Binary variable (%)** | |  | 34.1 | |  | 31.7 | |  | 30.5 | |  | 28.1 | |
