# Supplementary material for: Comparison of model-building strategies for excess hazard regression models in the context of cancer epidemiology
Source: BMC Med Res Methodol. 2019 Nov 20;19:210. doi: 10.1186/s12874-019-0830-9 (PMC6869178; doi:10.1186/s12874-019-0830-9)
Supplement: Supplementary file 4 — Additional file 4. Self-confounding and confounding effect due to mis-selection of variable effects (scenario A and C). [file 12874_2019_830_MOESM4_ESM.docx]

**Additional file 4**

Self-confounding and confounding effect due to mis-selection of variable effects (scenario A and C)

| **Scenario A** | |  |  |  |  |  |  |  |  | **Scenario C** | |  |  |  |  |  |  |  | |
| --- | --- | --- | --- | --- | --- | --- | --- | --- | --- | --- | --- | --- | --- | --- | --- | --- | --- | --- | --- |
|  | **Variables** | **MVRS** | | |  | **W&A** | | |  |  | **Variables** | **MVRS** | | |  | **W&A** | | |  |
|  |  | **p (%)** | **95% CI*** | |  | **p (%)** | **95% CI*** | |  |  |  | **p (%)** | **95% CI*** | |  | **p (%)** | **95% CI*** | | |
|  | Stage | 100.0 | 100.0 | 100.0 |  | 100.0 | 100.0 | 100.0 |  |  | Stage | 100.0 | 100.0 | 100.0 |  | 100.0 | 100.0 | 100.0 | |
|  | TD stage | 6.4 | 3.4 | 9.4 |  | 6.8 | 3.7 | 9.9 |  |  | TD stage | 96.8 | 94.7 | 99.0 |  | 100.0 | 100.0 | 100.0 | |
|  | Deprivation | 100.0 | 100.0 | 100.0 |  | 100.0 | 100.0 | 100.0 |  |  | Deprivation | 100.0 | 100.0 | 100.0 |  | 100.0 | 100.0 | 100.0 | |
|  | TD dep | 4.8 | 2.2 | 7.4 |  | 4.8 | 2.2 | 7.4 |  |  | TD dep | 6.0 | 3.1 | 8.9 |  | 4.4 | 1.9 | 6.9 | |
|  | Age | 92.4 | 89.2 | 95.6 |  | 92.8 | 89.7 | 96.0 |  |  | Age | 54.8 | 48.7 | 60.9 |  | 65.6 | 59.8 | 71.4 | |
|  | NL Age | 7.6 | 4.4 | 10.8 |  | 7.2 | 4.1 | 10.4 |  |  | NL Age | 45.2 | 39.1 | 51.3 |  | 34.4 | 28.6 | 40.2 | |
|  | TD age | 5.6 | 2.8 | 8.4 |  | 5.6 | 2.8 | 8.4 |  |  | TD age | 36.4 | 30.5 | 42.3 |  | 40.4 | 34.4 | 46.4 | |
|  | Bin. var. | 9.6 | 6.0 | 13.2 |  | 10.8 | 7.0 | 14.6 |  |  | Bin. var. | 8.8 | 5.3 | 12.3 |  | 7.2 | 4.1 | 10.4 | |
|  | TD bin. var. | 0.8 | -0.3 | 1.9 |  | 5.6 | 2.8 | 8.4 |  |  | TD bin. var. | 0.8 | -0.3 | 1.9 |  | 5.6 | 2.8 | 8.4 | |
|  |  |  |  |  |  |  |  |  |  |  |  |  |  |  |  |  |  |  | |
|  | **Overall model** |  |  |  |  |  |  |  |  |  | **Overall model** |  |  |  |  |  |  |  | |
|  | Contained | 100.0 | 100.0 | 100.0 |  | 100.0 | 100.0 | 100.0 |  |  | Contained | 10.8 | 7.0 | 14.6 |  | 6.0 | 3.1 | 8.9 | |
|  | correctly selected | 69.6 | 64.0 | 75.2 |  | 70.4 | 64.8 | 76.0 |  |  | correctly selected | 8.8 | 5.3 | 12.3 |  | 5.2 | 2.5 | 7.9 | |
|  |  |  |  |  |  |  |  |  |  |  |  |  |  |  |  |  |  |  | |
|  |  |  |  |  |  |  |  |  |  |  | almost correctly selected | |  |  |  |  |  |  | |
|  |  |  |  |  |  |  |  |  |  |  | L-TD age | 22.4 | 17.3 | 27.5 |  | 30.0 | 24.4 | 35.6 | |
|  |  |  |  |  |  |  |  |  |  |  | NL-PH age | 28.4 | 22.9 | 33.9 |  | 28.4 | 22.9 | 33.9 | |
|  |  |  |  |  |  |  |  |  |  |  | L-PH age | 23.2 | 18.1 | 28.4 |  | 28.0 | 22.5 | 33.5 | |
|  |  |  |  |  |  |  |  |  |  |  |  |  |  |  |  |  |  |  | |
|  |  |  |  |  |  |  |  |  |  |  | overall proportion of models nearly exactly selected | | | | | |  |  | |
|  |  |  |  |  |  |  |  |  |  |  |  |  | 82.8 |  |  |  | 91.6 |  | |

Underlined variables are in the original model

* formula for the 95% confidence intervals, with $z=1.96$ and$w=250$: $\frac{\hat{p}+\frac{z^{2}}{2w}}{1+\frac{z^{2}}{w}}\pm\frac{z}{1+\frac{z^{2}}{w}}\sqrt{\frac{\hat{p}(1-\hat{p})}{w}+\frac{z^{2}}{4w^{2}}}$, using the Wilson approximation(Wilson, 1927)

|  |  | **MVRS** | | | |  | | **W&A** | | | |  | |  |  | | **MVRS** | | | |  | | **W&A** | | | |
| --- | --- | --- | --- | --- | --- | --- | --- | --- | --- | --- | --- | --- | --- | --- | --- | --- | --- | --- | --- | --- | --- | --- | --- | --- | --- | --- |
| SCENARIO A | | **% model with the var** | | **% change with original** | |  | | **% model with the var** | | **% change with original** | |  | | SCENARIO C | | | **% model with the var** | | **% change with original** | |  | | **% model with the var** | | **% change with original** | |
| **TD age included** | | |  | |  | |  | |  | |  | | **TD age forgotten** | | |  | |  | |  | |  | |  | |  |
|  | Stage | 100.0 | | 0.0 | |  | | 100.0 | | 0.0 | |  | |  | Stage | | 100.0 | | 0.0 | |  | | 100.0 | | 0.0 | |
|  | TD stage | 7.1 | | 11.6 | |  | | 7.1 | | 5.0 | |  | |  | TD stage | | 95.0 | | -1.9 | |  | | 100.0 | | 0.0 | |
|  | Deprivation | 100.0 | | 0.0 | |  | | 100.0 | | 0.0 | |  | |  | Deprivation | | 100.0 | | 0.0 | |  | | 100.0 | | 0.0 | |
|  | TD dep | 0.0 | | -100.0 | |  | | 0.0 | | -100.0 | |  | |  | TD dep | | 5.7 | | -5.7 | |  | | 4.0 | | -8.5 | |
|  | Age | 92.9 | | 0.5 | |  | | 78.6 | | -15.3 | |  | |  | Age | | 45.9 | | -16.2 | |  | | 52.3 | | -20.2 | |
|  | NL Age | 7.1 | | -6.0 | |  | | 21.4 | | 197.6 | |  | |  | NL Age | | 54.1 | | 19.7 | |  | | 47.7 | | 38.5 | |
|  | *TD age* | *100.0* | |  | |  | | *100.0* | |  | |  | |  | *TD age* | | *0.0* | |  | |  | | *0.0* | |  | |
|  | bin. var | 7.1 | | -25.6 | |  | | 7.1 | | -33.9 | |  | |  | bin. var | | 8.8 | | 0.1 | |  | | 5.4 | | -25.4 | |
|  | TD bin. var | 0.0 | | -100.0 | |  | | 0.0 | | -100.0 | |  | |  | TD bin. var | | 0.6 | | -21.4 | |  | | 3.4 | | -40.1 | |
| **TD stage included** | | |  | |  | |  | |  | |  | | **TD stage forgotten** | | | | |  | |  | |  | |  | |  |
|  | Stage | 100.0 | | 0.0 | |  | | 100.0 | | 0.0 | |  | |  | Stage | | 100.0 | | 0.0 | |  | |  | |  | |
|  | *TD stage* | *100.0* | |  | |  | | *100.0* | |  | |  | |  | *TD stage* | | *0.0* | |  | |  | |  | |  | |
|  | Deprivation | 100.0 | | 0.0 | |  | | 100.0 | | 0.0 | |  | |  | Deprivation | | 100.0 | | 0.0 | |  | |  | |  | |
|  | TD dep | 12.5 | | 160.4 | |  | | 11.8 | | 145.1 | |  | |  | TD dep | | 0.0 | | -100.0 | |  | |  | |  | |
|  | Age | 87.5 | | -5.3 | |  | | 82.4 | | -11.3 | |  | |  | Age | | 0.0 | | -100.0 | |  | |  | |  | |
|  | NL Age | 12.5 | | 64.5 | |  | | 17.6 | | 145.1 | |  | |  | NL Age | | 100.0 | | 121.2 | |  | |  | |  | |
|  | TD age | 6.3 | | 11.6 | |  | | 5.9 | | 5.0 | |  | |  | TD age | | 0.0 | | -100.0 | |  | |  | |  | |
|  | bin. var | 6.3 | | -34.9 | |  | | 17.6 | | 63.4 | |  | |  | bin. var | | 12.5 | | 42.0 | |  | |  | |  | |
|  | TD bin. var | 0.0 | | -100.0 | |  | | 11.8 | | 110.1 | |  | |  | TD bin. var | | 0.0 | | -100.0 | |  | |  | |  | |
| **TD dep included** | | |  | |  | |  | |  | |  | | **TD dep included** | | |  | |  | |  | |  | |  | |  |
|  | Stage | 100.0 | | 0.0 | |  | | 100.0 | | 0.0 | |  | |  | Stage | | 100.0 | | 0.0 | |  | | 100.0 | | 0.0 | |
|  | TD stage | 16.7 | | 160.4 | |  | | 16.7 | | 145.1 | |  | |  | TD stage | | 100.0 | | 3.3 | |  | | 100.0 | | 0.0 | |
|  | Deprivation | 100.0 | | 0.0 | |  | | 100.0 | | 0.0 | |  | |  | Deprivation | | 100.0 | | 0.0 | |  | | 100.0 | | 0.0 | |
|  | *TD dep* | *100.0* | |  | |  | | *100.0* | |  | |  | |  | *TD dep* | | *100.0* | |  | |  | | *100.0* | |  | |
|  | Age | 100.0 | | 8.2 | |  | | 100.0 | | 7.8 | |  | |  | Age | | 53.3 | | -2.7 | |  | | 63.6 | | -3.0 | |
|  | NL Age | 0.0 | | -100.0 | |  | | 0.0 | | -100.0 | |  | |  | NL Age | | 46.7 | | 3.2 | |  | | 36.4 | | 5.7 | |
|  | TD age | 0.0 | | -100.0 | |  | | 0.0 | | -100.0 | |  | |  | TD age | | 40.0 | | 9.9 | |  | | 45.5 | | 12.5 | |
|  | bin. var | 0.0 | | -100.0 | |  | | 0.0 | | -100.0 | |  | |  | bin. var | | 6.7 | | -24.2 | |  | | 27.3 | | 278.8 | |
|  | TD bin. var | 0.0 | | -100.0 | |  | | 0.0 | | -100.0 | |  | |  | TD bin. var | | 0.0 | | -100.0 | |  | | 18.2 | | 224.7 | |
| **NL age instead of L age** | | |  | |  | |  | |  | |  | | **L age instead of NL age** | | | | |  | |  | |  | |  | |  |
|  | Stage | 100.0 | | 0.0 | |  | | 100.0 | | 0.0 | |  | |  | Stage | | 100.0 | | 0.0 | |  | | 100.0 | | 0.0 | |
|  | TD stage | 10.5 | | 64.5 | |  | | 16.7 | | 145.1 | |  | |  | TD stage | | 100.0 | | 3.3 | |  | | 100.0 | | 0.0 | |
|  | Deprivation | 100.0 | | 0.0 | |  | | 100.0 | | 0.0 | |  | |  | Deprivation | | 100.0 | | 0.0 | |  | | 100.0 | | 0.0 | |
|  | TD dep | 0.0 | | -100.0 | |  | | 0.0 | | -100.0 | |  | |  | TD dep | | 5.8 | | -2.7 | |  | | 4.3 | | -3.0 | |
|  | Age | 0.0 | | -100.0 | |  | | 0.0 | | -100.0 | |  | |  | Age | | 100.0 | | 82.5 | |  | | 100.0 | | 52.4 | |
|  | *NL Age* | *100.0* | |  | |  | | *100.0* | |  | |  | |  | *NL Age* | | *0.0* | |  | |  | | *0.0* | |  | |
|  | TD age | 5.3 | | -6.0 | |  | | 16.7 | | 197.6 | |  | |  | TD age | | 46.7 | | 28.3 | |  | | 52.4 | | 29.8 | |
|  | bin. var | 10.5 | | 9.6 | |  | | 16.7 | | 54.3 | |  | |  | bin. var | | 10.9 | | 24.4 | |  | | 7.9 | | 10.1 | |
|  | TD bin. var | 0.0 | | -100.0 | |  | | 11.1 | | 98.4 | |  | |  | TD bin. var | | 0.7 | | -8.8 | |  | | 7.3 | | 30.7 | |
| **Bin. var included** | | |  | |  | |  | |  | |  | | **Bin. var included** | | |  | |  | |  | |  | |  | |  |
|  | Stage | 100.0 | | 0.0 | |  | | 100.0 | | 0.0 | |  | |  | Stage | | 100.0 | | 0.0 | |  | | 100.0 | | 0.0 | |
|  | TD stage | 4.2 | | -34.9 | |  | | 11.1 | | 63.4 | |  | |  | TD stage | | 95.5 | | -1.4 | |  | | 100.0 | | 0.0 | |
|  | Deprivation | 100.0 | | 0.0 | |  | | 100.0 | | 0.0 | |  | |  | Deprivation | | 100.0 | | 0.0 | |  | | 100.0 | | 0.0 | |
|  | TD dep | 0.0 | | -100.0 | |  | | 0.0 | | -100.0 | |  | |  | TD dep | | 4.5 | | -24.2 | |  | | 16.7 | | 278.8 | |
|  | Age | 91.7 | | -0.8 | |  | | 88.9 | | -4.2 | |  | |  | Age | | 68.2 | | 24.4 | |  | | 72.2 | | 10.1 | |
|  | NL Age | 8.3 | | 9.6 | |  | | 11.1 | | 54.3 | |  | |  | NL Age | | 31.8 | | -29.6 | |  | | 27.8 | | -19.3 | |
|  | TD age | 4.2 | | -25.6 | |  | | 3.7 | | -33.9 | |  | |  | TD age | | 36.4 | | -0.1 | |  | | 55.6 | | 37.5 | |
|  | *bin. var* | 100.0 | |  | |  | | 100.0 | |  | |  | |  | *bin. var* | | 100.0 | |  | |  | | 100.0 | |  | |
|  | TD bin. var | 8.3 | | 941.7 | |  | | 51.9 | | 825.9 | |  | |  | TD bin. var | | 9.1 | | 1036.4 | |  | | 77.8 | | 1288.9 | |
| **TD Bin. var included** | | |  | |  | |  | |  | |  | | **TD Bin. var included** | | | | |  | |  | |  | |  | |  |
|  | Stage | 100.0 | | 0.0 | |  | | 100.0 | | 0.0 | |  | |  | Stage | | 100.0 | | 0.0 | |  | | 100.0 | | 0.0 | |
|  | TD stage | 0.0 | | -100.0 | |  | | 14.3 | | 110.1 | |  | |  | TD stage | | 100.0 | | 3.3 | |  | | 100.0 | | 0.0 | |
|  | Deprivation | 100.0 | | 0.0 | |  | | 100.0 | | 0.0 | |  | |  | Deprivation | | 100.0 | | 0.0 | |  | | 100.0 | | 0.0 | |
|  | TD dep | 0.0 | | -100.0 | |  | | 0.0 | | -100.0 | |  | |  | TD dep | | 0.0 | | -100.0 | |  | | 14.3 | | 224.7 | |
|  | Age | 100.0 | | 8.2 | |  | | 85.7 | | -7.6 | |  | |  | Age | | 50.0 | | -8.8 | |  | | 85.7 | | 30.7 | |
|  | NL Age | 0.0 | | -100.0 | |  | | 14.3 | | 98.4 | |  | |  | NL Age | | 50.0 | | 10.6 | |  | | 14.3 | | -58.5 | |
|  | TD age | 0.0 | | -100.0 | |  | | 0.0 | | -100.0 | |  | |  | TD age | | 50.0 | | 37.4 | |  | | 64.3 | | 59.1 | |
|  | Bin. var | 100.0 | | 941.7 | |  | | 100.0 | | 825.9 | |  | |  | Bin. var | | 100.0 | | 1036.4 | |  | | 100.0 | | 1288.9 | |
|  | *TD Bin. var* | *100.0* | |  | |  | | *100.0* | |  | |  | |  | *TD Bin. var* | | *100.0* | |  | |  | | *100.0* | |  | |

Self-confounding and confounding effect due to mis-selection of variable effects (scenario B and D)

| **Scenario B** | |  |  |  |  |  | |  | |  |  |  |  |  |  | **Scenario D** | |  |  |  |  |  |  |  |  |  |  |  |
| --- | --- | --- | --- | --- | --- | --- | --- | --- | --- | --- | --- | --- | --- | --- | --- | --- | --- | --- | --- | --- | --- | --- | --- | --- | --- | --- | --- | --- |
|  | **Variables** | **aMVRS** | | |  | | **MFPIgen** | | | |  | **aW&A** | | |  |  | **Variables** | **aMVRS** | | |  | **MFPIgen** | | |  | **aW&A** | | |
|  |  | **p (%)** | **95% CI*** | |  | | **p (%)** | | **95% CI*** | |  | **p (%)** | **95% CI*** | |  |  |  | **p (%)** | **95% CI*** | |  | **p (%)** | **95% CI*** | |  | **p (%)** | **95% CI*** | |
|  | Stage | 100.0 | 100.0 | 100.0 |  | 100.0 | | 100.0 | | 100.0 |  | 100.0 | 100.0 | 100.0 |  |  | Stage | 100.0 | 100.0 | 100.0 |  | 100.0 | 100.0 | 100.0 |  | 100.0 | 100.0 | 100.0 |
|  | TD stage | 6.0 | 3.1 | 8.9 |  | 6.8 | | 3.7 | | 9.9 |  | 9.2 | 5.7 | 12.7 |  |  | TD stage | 100.0 | 100.0 | 100.0 |  | 99.2 | 98.1 | 100.3 |  | 99.6 | 98.8 | 100.4 |
|  | Deprivation | 100.0 | 100.0 | 100.0 |  | 100.0 | | 100.0 | | 100.0 |  | 100.0 | 100.0 | 100.0 |  |  | Deprivation | 100.0 | 100.0 | 100.0 |  | 100.0 | 100.0 | 100.0 |  | 100.0 | 100.0 | 100.0 |
|  | TD dep | 4.8 | 2.2 | 7.4 |  | 3.6 | | 1.3 | | 5.9 |  | 6.8 | 3.7 | 9.9 |  |  | TD dep | 10.0 | 6.3 | 13.7 |  | 6.0 | 3.1 | 8.9 |  | 18.4 | 13.7 | 23.1 |
|  | Age | 88.8 | 85.0 | 92.7 |  | 94.0 | | 91.1 | | 96.9 |  | 87.6 | 83.6 | 91.6 |  |  | Age | 66.4 | 60.6 | 72.2 |  | 60.4 | 54.4 | 66.4 |  | 72.4 | 67.0 | 77.9 |
|  | NL Age | 11.2 | 7.4 | 15.1 |  | 6.0 | | 3.1 | | 8.9 |  | 12.4 | 8.4 | 16.4 |  |  | NL Age | 33.6 | 27.8 | 39.4 |  | 39.6 | 33.6 | 45.6 |  | 27.6 | 22.2 | 33.1 |
|  | TD age | 7.6 | 4.4 | 10.8 |  | 6.4 | | 3.4 | | 9.4 |  | 5.6 | 2.8 | 8.4 |  |  | TD age | 48.0 | 41.9 | 54.1 |  | 36.0 | 30.1 | 41.9 |  | 28.8 | 23.3 | 34.3 |
|  | Bin. var. | 23.2 | 18.1 | 28.4 |  | 11.6 | | 7.7 | | 15.5 |  | 17.2 | 12.6 | 21.8 |  |  | Bin. var. | 26.4 | 21.0 | 31.8 |  | 16.8 | 12.2 | 21.4 |  | 20.8 | 15.9 | 25.8 |
|  | TD bin. var. | 1.6 | 0.1 | 3.1 |  | 0.4 | | -0.4 | | 1.2 |  | 6.4 | 3.4 | 9.4 |  |  | TD bin. var. | 3.6 | 1.3 | 5.9 |  | 1.6 | 0.1 | 3.1 |  | 9.2 | 5.7 | 12.7 |
|  | A*stage | 35.6 | 29.8 | 41.5 |  | 29.6 | | 24.0 | | 35.2 |  | 35.2 | 29.4 | 41.0 |  |  | A*stage | 28.4 | 22.9 | 33.9 |  | 50.8 | 44.7 | 56.9 |  | 70.8 | 65.3 | 76.4 |
|  | TD A*stage | 0.4 | -0.4 | 1.2 |  | 0.0 | | 0.0 | | 0.0 |  | 4.0 | 1.6 | 6.4 |  |  | TD A*stage | 1.6 | 0.1 | 3.1 |  | 0.0 | 0.0 | 0.0 |  | 22.8 | 17.7 | 27.9 |
|  | A*dep | 11.6 | 7.7 | 15.5 |  | 4.8 | | 2.2 | | 7.4 |  | 10.0 | 6.3 | 13.7 |  |  | A*dep | 16.0 | 11.5 | 20.5 |  | 6.0 | 3.1 | 8.9 |  | 21.2 | 16.2 | 26.2 |
|  | TD A*dep | 1.2 | -0.1 | 2.5 |  | 0.0 | | 0.0 | | 0.0 |  | 3.2 | 1.1 | 5.4 |  |  | TD A*dep | 3.6 | 1.3 | 5.9 |  | 0.0 | 0.0 | 0.0 |  | 14.0 | 9.8 | 18.2 |
|  | A*bin. var. | 11.2 | 7.4 | 15.1 |  | 2.4 | | 0.5 | | 4.3 |  | 10.8 | 7.0 | 14.6 |  |  | A*bin. var. | 14.4 | 10.1 | 18.7 |  | 4.8 | 2.2 | 7.4 |  | 11.6 | 7.7 | 15.5 |
|  | TD A*bin. var. | 0.4 | -0.4 | 1.2 |  | 0.0 | | 0.0 | | 0.0 |  | 2.8 | 0.8 | 4.8 |  |  | TD A*bin. var. | 0.4 | -0.4 | 1.2 |  | 0.0 | 0.0 | 0.0 |  | 3.6 | 1.3 | 5.9 |
|  |  |  |  |  |  |  | |  | |  |  |  |  |  |  |  |  |  |  |  |  |  |  |  |  |  |  |  |
|  | **Overall model** |  |  |  |  |  | |  | |  |  |  |  |  |  |  | **Overall model** | | |  |  |  |  |  |  |  |  |  |
|  | Contained | 35.6 | 29.8 | 41.5 |  | 29.6 | | 24.0 | | 35.2 |  | 35.2 | 29.4 | 41.0 |  |  | Contained | 2.4 | 0.5 | 4.3 |  | 3.2 | 1.1 | 5.4 |  | 4.8 | 2.2 | 7.4 |
|  | Correctly selected | 14.4 | 10.1 | 18.7 |  | 14.4 | | 10.1 | | 18.7 |  | 14.8 | 10.5 | 19.1 |  |  | Correctly selected | 1.6 | 0.1 | 3.1 |  | 2.8 | 0.8 | 4.8 |  | 1.6 | 0.1 | 3.1 |
|  |  |  |  |  |  |  | |  | |  |  |  |  |  |  |  |  |  |  |  |  |  |  |  |  |  |  |  |
|  | Almost correctly selected | |  |  |  |  | |  | |  |  |  |  |  |  |  | Almost correctly selected | | | | |  |  |  |  |  |  |  |
|  | no interaction | 39.2 | 33.2 | 45.2 |  | 52.4 | | 46.3 | | 58.5 |  | 45.2 | 39.1 | 51.3 |  |  | L Age | 9.6 | 6.0 | 13.2 |  | 13.6 | 9.4 | 17.8 |  | 10.4 | 6.7 | 14.1 |
|  |  |  |  |  |  |  | |  | |  |  |  |  |  |  |  | NL-PH Age | 0.8 | -0.3 | 1.9 |  | 8.4 | 5.0 | 11.8 |  | 2.4 | 0.5 | 4.3 |
|  | overall proportion of models nearly exactly selected | | | | | | | | | | | | |  |  |  | L-PH Age | 4.0 | 1.6 |  |  | 12.0 | 6.4 | 16.0 |  | 8.8 | 5.3 | 12.3 |
|  |  | 53.6 | 47.5 | 59.7 |  | 66.8 | | 61.1 | | 72.6 |  | 60.0 | 54.0 | 66.0 |  |  | no A*stage (NL-TD age) | 4.0 | 1.6 | 6.4 |  | 4.4 | 1.9 | 6.9 |  | 1.2 | -0.1 | 2.5 |
|  |  |  |  |  |  |  | |  | |  |  |  |  |  |  |  | no A*stage (NL-PH age) | 14.0 | 9.8 | 18.2 |  | 13.2 | 9.1 | 17.3 |  | 4.8 | 2.2 | 7.4 |
|  |  |  |  |  |  |  | |  | |  |  |  |  |  |  |  | no A*stage (L-TD age) | 11.2 | 7.4 | 15.1 |  | 6.4 | 3.4 | 9.4 |  | 3.6 | 1.3 | 5.9 |
|  |  |  |  |  |  |  | |  | |  |  |  |  |  |  |  | no A*stage (L-PH age) | 14.4 | 10.1 | 18.7 |  | 12.0 | 8.0 | 16.0 |  | 8.8 | 5.3 | 12.3 |
|  |  |  |  |  |  |  | |  | |  |  |  |  |  |  |  |  |  |  |  |  |  |  |  |  |  |  |  |
|  |  |  |  |  |  |  | |  | |  |  |  |  |  |  |  | overall proportion of models nearly exactly selected | | | | | | |  |  |  |  |  |
|  |  |  |  |  |  |  | |  | |  |  |  |  |  |  |  |  | 59.6 | 53.6 | 65.6 |  | 72.8 | 67.4 | 78.2 |  | 41.6 | 35.6 | 47.6 |
|  |  |  |  |  |  |  | |  | |  |  |  |  |  |  |  | overall proportion of models nearly exactly selected (WITH interaction) | | | | | | | | | | | |
|  |  |  |  |  |  |  | |  | |  |  |  |  |  |  |  |  | 16.0 | 11.5 | 20.5 |  | 36.8 | 30.9 | 42.7 |  | 23.2 | 18.1 | 28.4 |

Underlined variables are in the original model

* formula for the 95% confidence intervals, with $z=1.96$ and$w=250$: $\frac{\hat{p}+\frac{z^{2}}{2w}}{1+\frac{z^{2}}{w}}\pm\frac{z}{1+\frac{z^{2}}{w}}\sqrt{\frac{\hat{p}(1-\hat{p})}{w}+\frac{z^{2}}{4w^{2}}}$, using the Wilson approximation(Wilson, 1927)

|  | |  | **aMVRS** | |  | **MFPIgen** | |  | **aW&A** | |  |
| --- | --- | --- | --- | --- | --- | --- | --- | --- | --- | --- | --- |
| SCENARIO B | | | **% model with the var** | **% change with original** |  | **% model with the var** | **% change with original** |  | **% model with the var** | **% change with original** |  |
| **TD age included** | | |  |  |  |  |  |  |  |  |  |
|  | | Stage | 100.0 | 0.0 |  | 100.0 | 0.0 |  | 100.0 | 0.0 |  |
|  | | TD stage | 21.1 | 250.9 |  | 12.5 | 83.8 |  | 28.6 | 210.6 |  |
|  | | Deprivation | 100.0 | 0.0 |  | 100.0 | 0.0 |  | 100.0 | 0.0 |  |
|  | | TD dep | 5.3 | 9.6 |  | 6.3 | 73.6 |  | 0.0 | -100.0 |  |
|  | | Age | 84.2 | -5.2 |  | 93.8 | -0.3 |  | 71.4 | -18.5 |  |
|  | | NL Age | 15.8 | 41.0 |  | 6.3 | 4.2 |  | 28.6 | 130.4 |  |
|  | | *TD age* | *100.0* |  |  | *100.0* |  |  | *100.0* |  |  |
|  | | Bin. var | 15.8 | -31.9 |  | 6.3 | -46.1 |  | 14.3 | -16.9 |  |
|  | | TD bin. var | 10.5 | 557.9 |  | 0.0 | -100.0 |  | 7.1 | 11.6 |  |
|  | | A*stage | 36.8 | 3.5 |  | 18.8 | -36.7 |  | 42.9 | 21.8 |  |
|  | | TD A*stage | 0.0 | -100.0 |  | 0.0 |  |  | 14.3 | 257.1 |  |
|  | | A*dep | 10.5 | -9.3 |  | 0.0 | -100.0 |  | 14.3 | 42.9 |  |
|  | | TD A*dep | 0.0 | -100.0 |  | 0.0 |  |  | 0.0 | -100.0 |  |
|  | | A*bin. var | 15.8 | 41.0 |  | 6.3 | 160.4 |  | 14.3 | 32.3 |  |
|  | | TD A*bin. var | 5.3 | 1215.8 |  | 0.0 |  |  | 7.1 | 155.1 |  |
| **TD stage included** | | |  |  |  |  |  |  |  |  |  |
|  | | Stage | 100.0 | 0.0 |  | 100.0 | 0.0 |  | 100.0 | 0.0 |  |
|  | | *TD stage* | *100.0* |  |  | *100.0* |  |  | *100.0* |  |  |
|  | | Deprivation | 100.0 | 0.0 |  | 100.0 | 0.0 |  | 100.0 | 0.0 |  |
|  | | TD dep | 13.3 | 177.8 |  | 5.9 | 63.4 |  | 17.4 | 155.8 |  |
|  | | Age | 73.3 | -17.4 |  | 94.1 | 0.1 |  | 69.6 | -20.6 |  |
|  | | NL Age | 26.7 | 138.1 |  | 5.9 | -2.0 |  | 30.4 | 145.4 |  |
|  | | TD age | 26.7 | 250.9 |  | 11.8 | 83.8 |  | 17.4 | 210.6 |  |
|  | | Bin. var | 13.3 | -42.5 |  | 11.8 | 1.4 |  | 8.7 | -49.4 |  |
|  | | TD bin. var | 6.7 | 316.7 |  | 0.0 | -100.0 |  | 8.7 | 35.9 |  |
|  | | A*stage | 60.0 | 68.5 |  | 58.8 | 98.7 |  | 73.9 | 110.0 |  |
|  | | TD A*stage | 6.7 | 1566.7 |  | 0.0 |  |  | 43.5 | 987.0 |  |
|  | | A*dep | 26.7 | 129.9 |  | 0.0 | -100.0 |  | 21.7 | 117.4 |  |
|  | | TD A*dep | 6.7 | 455.6 |  | 0.0 |  |  | 13.0 | 307.6 |  |
|  | | A*bin. var | 6.7 | -40.5 |  | 5.9 | 145.1 |  | 8.7 | -19.5 |  |
|  | | TD A*bin. var | 0.0 | -100.0 |  | 0.0 |  |  | 4.3 | 55.3 |  |
| **TD dep included** | | |  |  |  |  |  |  |  |  |  |
|  | | Stage | 100.0 | 0.0 |  | 100.0 | 0.0 |  | 100.0 | 0.0 |  |
|  | | TD stage | 16.7 | 177.8 |  | 11.1 | 63.4 |  | 23.5 | 155.8 |  |
|  | | Deprivation | 100.0 | 0.0 |  | 100.0 | 0.0 |  | 100.0 | 0.0 |  |
|  | | *TD dep* | *100.0* |  |  | *100.0* |  |  | *100.0* |  |  |
|  | | Age | 91.7 | 3.2 |  | 88.9 | -5.4 |  | 76.5 | -12.7 |  |
|  | | NL Age | 8.3 | -25.6 |  | 11.1 | 85.2 |  | 23.5 | 89.8 |  |
|  | | TD age | 8.3 | 9.6 |  | 11.1 | 73.6 |  | 0.0 | -100.0 |  |
|  | | Bin. var | 16.7 | -28.2 |  | 0.0 | -100.0 |  | 29.4 | 71.0 |  |
|  | | TD bin. var | 0.0 | -100.0 |  | 0.0 | -100.0 |  | 23.5 | 267.6 |  |
|  | | A*stage | 41.7 | 17.0 |  | 33.3 | 12.6 |  | 35.3 | 0.3 |  |
|  | | TD A*stage | 8.3 | 1983.3 |  | 0.0 |  |  | 17.6 | 341.2 |  |
|  | | A*dep | 33.3 | 187.4 |  | 0.0 | -100.0 |  | 52.9 | 429.4 |  |
|  | | TD A*dep | 25.0 | 1983.3 |  | 0.0 |  |  | 47.1 | 1370.6 |  |
|  | | A*bin. var | 16.7 | 48.8 |  | 0.0 | -100.0 |  | 11.8 | 8.9 |  |
|  | | TD A*bin. var | 0.0 | -100.0 |  | 0.0 |  |  | 5.9 | 110.1 |  |
| **NL age instead of L age** | | | |  |  |  |  |  |  |  |  |
|  | | Stage | 100.0 | 0.0 |  | 100.0 | 0.0 |  | 100.0 | 0.0 |  |
|  | | TD stage | 14.3 | 138.1 |  | 6.7 | -2.0 |  | 22.6 | 145.4 |  |
|  | | Deprivation | 100.0 | 0.0 |  | 100.0 | 0.0 |  | 100.0 | 0.0 |  |
|  | | TD dep | 3.6 | -25.6 |  | 6.7 | 85.2 |  | 12.9 | 89.8 |  |
|  | | Age | 0.0 | -100.0 |  | 0.0 | -100.0 |  | 0.0 | -100.0 |  |
|  | | *NL Age* | *100.0* |  |  | *100.0* |  |  | *100.0* |  |  |
|  | | TD age | 10.7 | 41.0 |  | 6.7 | 4.2 |  | 12.9 | 130.4 |  |
|  | | Bin. var | 32.1 | 38.5 |  | 13.3 | 14.9 |  | 32.3 | 87.5 |  |
|  | | TD bin. var | 0.0 | -100.0 |  | 0.0 | -100.0 |  | 12.9 | 101.6 |  |
|  | | A*stage | 35.7 | 0.3 |  | 20.0 | -32.4 |  | 48.4 | 37.5 |  |
|  | | TD A*stage | 0.0 | -100.0 |  | 0.0 |  |  | 12.9 | 222.6 |  |
|  | | A*dep | 21.4 | 84.7 |  | 0.0 | -100.0 |  | 19.4 | 93.5 |  |
|  | | TD A*dep | 0.0 | -100.0 |  | 0.0 |  |  | 6.5 | 101.6 |  |
|  | | A*bin. var | 21.4 | 91.3 |  | 0.0 | -100.0 |  | 29.0 | 168.8 |  |
|  | | TD A*bin. var | 0.0 | -100.0 |  | 0.0 |  |  | 9.7 | 245.6 |  |
| **CScore included** | | |  |  |  |  |  |  |  |  |  |
|  | | Stage | 100.0 | 0.0 |  | 100.0 | 0.0 |  | 100.0 | 0.0 |  |
|  | | TD stage | 3.4 | -42.5 |  | 6.9 | 1.4 |  | 4.7 | -49.4 |  |
|  | | Deprivation | 100.0 | 0.0 |  | 100.0 | 0.0 |  | 100.0 | 0.0 |  |
|  | | TD dep | 3.4 | -28.2 |  | 0.0 | -100.0 |  | 11.6 | 71.0 |  |
|  | | Age | 84.5 | -4.9 |  | 93.1 | -1.0 |  | 76.7 | -12.4 |  |
|  | | NL Age | 15.5 | 38.5 |  | 6.9 | 14.9 |  | 23.3 | 87.5 |  |
|  | | TD age | 5.2 | -31.9 |  | 3.4 | -46.1 |  | 4.7 | -16.9 |  |
|  | | *Bin. var* | *100.0* |  |  | *100.0* |  |  | *100.0* |  |  |
|  | | TD bin. var | 6.9 | 331.0 |  | 3.4 | 762.1 |  | 37.2 | 481.4 |  |
|  | | A*stage | 41.4 | 16.2 |  | 58.6 | 98.0 |  | 34.9 | -0.9 |  |
|  | | TD A*stage | 0.0 | -100.0 |  | 0.0 |  |  | 2.3 | -41.9 |  |
|  | | A*dep | 10.3 | -10.8 |  | 10.3 | 115.5 |  | 9.3 | -7.0 |  |
|  | | TD A*dep | 0.0 | -100.0 |  | 0.0 |  |  | 2.3 | -27.3 |  |
|  | | A*bin. var | 48.3 | 331.0 |  | 20.7 | 762.1 |  | 62.8 | 481.4 |  |
|  | | TD A*bin. var | 1.7 | 331.0 |  | 0.0 |  |  | 16.3 | 481.4 |  |
| **TD CScore included** | | | |  |  |  |  |  |  |  |  |
|  | | Stage | 100.0 | 0.0 |  | 100.0 | 0.0 |  | 100.0 | 0.0 |  |
|  | | TD stage | 25.0 | 316.7 |  | 0.0 | -100.0 |  | 12.5 | 35.9 |  |
|  | | Deprivation | 100.0 | 0.0 |  | 100.0 | 0.0 |  | 100.0 | 0.0 |  |
|  | | TD dep | 0.0 | -100.0 |  | 0.0 | -100.0 |  | 25.0 | 267.6 |  |
|  | | Age | 100.0 | 12.6 |  | 100.0 | 6.4 |  | 75.0 | -14.4 |  |
|  | | NL Age | 0.0 | -100.0 |  | 0.0 | -100.0 |  | 25.0 | 101.6 |  |
|  | | TD age | 50.0 | 557.9 |  | 0.0 | -100.0 |  | 6.3 | 11.6 |  |
|  | | Bin. var | 100.0 | 331.0 |  | 100.0 | 762.1 |  | 100.0 | 481.4 |  |
|  | | *TD bin. var* | *100.0* |  |  | *100.0* |  |  | *100.0* |  |  |
|  | | A*stage | 25.0 | -29.8 |  | 0.0 | -100.0 |  | 18.8 | -46.7 |  |
|  | | TD A*stage | 0.0 | -100.0 |  | 0.0 |  |  | 6.3 | 56.3 |  |
|  | | A*dep | 25.0 | 115.5 |  | 0.0 | -100.0 |  | 12.5 | 25.0 |  |
|  | | TD A*dep | 0.0 | -100.0 |  | 0.0 |  |  | 6.3 | 95.3 |  |
|  | | A*bin. var | 75.0 | 569.6 |  | 0.0 | -100.0 |  | 56.3 | 420.8 |  |
|  | | TD A*bin. var | 25.0 | 6150.0 |  | 0.0 |  |  | 43.8 | 1462.5 |  |
| **A*dep included** | | |  |  |  |  |  |  |  |  |  |
|  | | Stage | 100.0 | 0.0 |  | 100.0 | 0.0 |  | 100.0 | 0.0 |  |
|  | | TD stage | 13.8 | 129.9 |  | 0.0 | -100.0 |  | 20.0 | 117.4 |  |
|  | | Deprivation | 100.0 | 0.0 |  | 100.0 | 0.0 |  | 100.0 | 0.0 |  |
|  | | TD dep | 13.8 | 187.4 |  | 0.0 | -100.0 |  | 36.0 | 429.4 |  |
|  | | Age | 79.3 | -10.7 |  | 100.0 | 6.4 |  | 76.0 | -13.2 |  |
|  | | NL Age | 20.7 | 84.7 |  | 0.0 | -100.0 |  | 24.0 | 93.5 |  |
|  | | TD age | 6.9 | -9.3 |  | 0.0 | -100.0 |  | 8.0 | 42.9 |  |
|  | | Bin. var | 20.7 | -10.8 |  | 25.0 | 115.5 |  | 16.0 | -7.0 |  |
|  | | TD bin. var | 3.4 | 115.5 |  | 0.0 | -100.0 |  | 8.0 | 25.0 |  |
|  | | A*stage | 51.7 | 45.3 |  | 75.0 | 153.4 |  | 56.0 | 59.1 |  |
|  | | TD A*stage | 0.0 | -100.0 |  | 0.0 |  |  | 8.0 | 100.0 |  |
|  | | *A*dep* | *100.0* |  |  | *100.0* |  |  | *100.0* |  |  |
|  | | TD A*dep | 10.3 | 762.1 |  | 0.0 |  |  | 32.0 | 900.0 |  |
|  | | A*bin. var | 10.3 | -7.6 |  | 8.3 | 247.2 |  | 8.0 | -25.9 |  |
|  | | TD A*bin. var | 0.0 | -100.0 |  | 0.0 |  |  | 0.0 | -100.0 |  |
| **A*stage missed** | | |  |  |  |  |  |  |  |  |  |
|  | | Stage | 100.0 | 0.0 |  | 100.0 | 0.0 |  | 100.0 | 0.0 |  |
|  | | TD stage | 3.7 | -37.9 |  | 4.0 | -41.5 |  | 3.7 | -59.7 |  |
|  | | Deprivation | 100.0 | 0.0 |  | 100.0 | 0.0 |  | 100.0 | 0.0 |  |
|  | | TD dep | 4.3 | -9.4 |  | 3.4 | -5.3 |  | 6.8 | -0.1 |  |
|  | | Age | 88.8 | 0.0 |  | 93.2 | -0.9 |  | 90.1 | 2.9 |  |
|  | | NL Age | 11.2 | -0.2 |  | 6.8 | 13.6 |  | 9.9 | -20.4 |  |
|  | | TD age | 7.5 | -1.9 |  | 7.4 | 15.4 |  | 4.9 | -11.8 |  |
|  | | Bin. var | 21.1 | -9.0 |  | 6.8 | -41.2 |  | 17.3 | 0.5 |  |
|  | | TD bin. var | 1.9 | 16.5 |  | 0.6 | 42.0 |  | 8.0 | 25.4 |  |
|  | | *A*stage* | *0.0* |  |  | *0.0* |  |  | *0.0* |  |  |
|  | | TD A*stage | 0.0 | -100.0 |  | 0.0 |  |  | 0.0 | -100.0 |  |
|  | | A*dep | 8.7 | -25.0 |  | 1.7 | -64.5 |  | 6.8 | -32.1 |  |
|  | | TD A*dep | 0.6 | -48.2 |  | 0.0 |  |  | 3.1 | -3.5 |  |
|  | | A*bin. var | 10.6 | -5.7 |  | 0.0 | -100.0 |  | 9.9 | -8.6 |  |
|  | | TD A*bin. var | 0.6 | 55.3 |  | 0.0 |  |  | 2.5 | -11.8 |  |
|  |  | | **aMVRS** | |  | **MFPIgen** | |  | **aW&A** | |  |
| SCENARIO D | | | **% model with the var** | **% change with original** |  | **% model with the var** | **% change with original** |  | **% model with the var** | **% change with original** |  |
| **Dd - TD age forgotten** | | |  |  |  |  |  |  |  |  |  |
|  | Stage | | 100.0 | 0.0 |  | 100.0 | 0.0 |  | 100.0 | 0.0 |  |
|  | TD stage | | 100.0 | 0.0 |  | 98.8 | -0.5 |  | 99.4 | -0.2 |  |
|  | Deprivation | | 100.0 | 0.0 |  | 100.0 | 0.0 |  | 100.0 | 0.0 |  |
|  | TD dep | | 8.5 | -15.4 |  | 5.6 | -6.3 |  | 20.8 | 13.0 |  |
|  | Age | | 53.1 | -20.1 |  | 52.5 | -13.1 |  | 71.3 | -1.5 |  |
|  | NL Age | | 46.9 | 39.7 |  | 47.5 | 19.9 |  | 28.7 | 3.8 |  |
|  | *TD age* | | *0.0* |  |  | *0.0* |  |  | *0.0* |  |  |
|  | Bin. var | | 25.4 | -3.8 |  | 19.4 | 15.3 |  | 20.8 | -0.1 |  |
|  | TD bin. var | | 3.1 | -14.5 |  | 1.9 | 17.2 |  | 8.4 | -8.4 |  |
|  | A*stage | | 16.2 | -43.1 |  | 45.6 | -10.2 |  | 69.7 | -1.6 |  |
|  | TD A*stage | | 0.8 | -51.9 |  | 0.0 |  |  | 30.3 | 33.1 |  |
|  | A*dep | | 12.3 | -23.1 |  | 5.0 | -16.7 |  | 24.7 | 16.6 |  |
|  | TD A*dep | | 2.3 | -35.9 |  | 0.0 |  |  | 18.0 | 28.4 |  |
|  | A*bin. var | | 11.5 | -19.9 |  | 5.6 | 17.2 |  | 10.7 | -8.0 |  |
|  | TD A*bin. var | | 0.8 | 92.3 |  | 0.0 |  |  | 2.8 | -22.0 |  |
| **Da - TD stage forgotten** | | | |  |  |  |  |  |  |  |  |
|  | Stage | |  |  |  | 100.0 | 0.0 |  | 100.0 | 0 |  |
|  | *TD stage* | |  |  |  | *0.0* |  |  | *0.0* |  |  |
|  | Deprivation | |  |  |  | 100.0 | 0.0 |  | 100.0 | 0 |  |
|  | TD dep | |  |  |  | 0.0 | -100.0 |  | 0.0 | -100 |  |
|  | Age | |  |  |  | 0.0 | -100.0 |  | 0.0 | -100 |  |
|  | NL Age | |  |  |  | 100.0 | 152.5 |  | 100.0 | 262.31884 |  |
|  | TD age | |  |  |  | 0.0 | -100.0 |  | 0.0 | -100 |  |
|  | Bin. var | |  |  |  | 0.0 | -100.0 |  | 100.0 | 380.76923 |  |
|  | TD bin. var | |  |  |  | 0.0 | -100.0 |  | 0.0 | -100 |  |
|  | A*stage | |  |  |  | 0.0 | -100.0 |  | 0.0 | -100 |  |
|  | TD A*stage | |  |  |  | 0.0 |  |  | 0.0 | -100 |  |
|  | A*dep | |  |  |  | 0.0 | -100.0 |  | 0.0 | -100 |  |
|  | TD A*dep | |  |  |  | 0.0 |  |  | 0.0 | -100 |  |
|  | A*bin. var | |  |  |  | 0.0 | -100.0 |  | 0.0 | -100 |  |
|  | TD A*bin. var | |  |  |  | 0.0 |  |  | 0.0 | -100 |  |
| **Db - TD dep included** | | |  |  |  |  |  |  |  |  |  |
|  | Stage | | 100.0 | 0.0 |  | 100.0 | 0.0 |  | 100.0 | 0.0 |  |
|  | TD stage | | 100.0 | 0.0 |  | 100.0 | 0.8 |  | 100.0 | 0.4 |  |
|  | Deprivation | | 100.0 | 0.0 |  | 100.0 | 0.0 |  | 100.0 | 0.0 |  |
|  | *TD dep* | | *100.0* |  |  | *100.0* |  |  | *100.0* |  |  |
|  | Age | | 68.0 | 2.4 |  | 60.0 | -0.7 |  | 78.3 | 8.1 |  |
|  | NL Age | | 32.0 | -4.8 |  | 40.0 | 1.0 |  | 21.7 | -21.2 |  |
|  | TD age | | 56.0 | 16.7 |  | 40.0 | 11.1 |  | 19.6 | -32.1 |  |
|  | Bin. var | | 28.0 | 6.1 |  | 13.3 | -20.6 |  | 13.0 | -37.3 |  |
|  | TD bin. var | | 8.0 | 122.2 |  | 6.7 | 316.7 |  | 6.5 | -29.1 |  |
|  | A*stage | | 20.0 | -29.6 |  | 46.7 | -8.1 |  | 78.3 | 10.5 |  |
|  | TD A*stage | | 0.0 | -100.0 |  | 0.0 |  |  | 6.5 | -71.4 |  |
|  | A*dep | | 60.0 | 275.0 |  | 6.7 | 11.1 |  | 78.3 | 269.2 |  |
|  | TD A*dep | | 36.0 | 900.0 |  | 0.0 |  |  | 76.1 | 443.5 |  |
|  | A*bin. var | | 24.0 | 66.7 |  | 0.0 | -100.0 |  | 8.7 | -25.0 |  |
|  | TD A*bin. var | | 0.0 | -100.0 |  | 0.0 |  |  | 2.2 | -39.6 |  |
| **Dc - L age instead of NL age** | | | |  |  |  |  |  |  |  |  |
|  | Stage | | 100.0 | 0.0 |  | 100.0 | 0.0 |  | 100.0 | 0.0 |  |
|  | TD stage | | 100.0 | 0.0 |  | 100.0 | 0.8 |  | 100.0 | 0.4 |  |
|  | Deprivation | | 100.0 | 0.0 |  | 100.0 | 0.0 |  | 100.0 | 0.0 |  |
|  | TD dep | | 10.2 | 2.4 |  | 6.0 | -0.7 |  | 19.9 | 8.1 |  |
|  | Age | | 100.0 | 50.6 |  | 100.0 | 65.6 |  | 100.0 | 38.1 |  |
|  | *NL Age* | | *0.0* |  |  | *0.0* |  |  | *0.0* |  |  |
|  | TD age | | 58.4 | 21.7 |  | 44.4 | 23.3 |  | 29.8 | 3.6 |  |
|  | Bin. var | | 25.9 | -1.9 |  | 16.6 | -1.5 |  | 18.2 | -12.3 |  |
|  | TD bin. var | | 3.0 | -16.3 |  | 0.7 | -58.6 |  | 8.8 | -3.9 |  |
|  | A*stage | | 36.1 | 27.3 |  | 60.3 | 18.6 |  | 74.6 | 5.3 |  |
|  | TD A*stage | | 1.8 | 13.0 |  | 0.0 |  |  | 22.1 | -3.1 |  |
|  | A*dep | | 16.9 | 5.4 |  | 5.3 | -11.7 |  | 21.5 | 1.6 |  |
|  | TD A*dep | | 4.8 | 33.9 |  | 0.0 |  |  | 16.6 | 18.4 |  |
|  | A*bin. var | | 14.5 | 0.4 |  | 5.3 | 10.4 |  | 9.4 | -19.0 |  |
|  | TD A*bin. var | | 0.0 | -100.0 |  | 0.0 |  |  | 3.3 | -7.9 |  |
| **De - Cscore included** | | |  |  |  |  |  |  |  |  |  |
|  | Stage | | 100.0 | 0.0 |  | 100.0 | 0.0 |  | 100.0 | 0.0 |  |
|  | TD stage | | 100.0 | 0.0 |  | 100.0 | 0.8 |  | 98.1 | -1.5 |  |
|  | Deprivation | | 100.0 | 0.0 |  | 100.0 | 0.0 |  | 100.0 | 0.0 |  |
|  | TD dep | | 10.6 | 6.1 |  | 4.8 | -20.6 |  | 11.5 | -37.3 |  |
|  | Age | | 65.2 | -1.9 |  | 59.5 | -1.5 |  | 63.5 | -12.3 |  |
|  | NL Age | | 34.8 | 3.7 |  | 40.5 | 2.2 |  | 36.5 | 32.4 |  |
|  | TD age | | 50.0 | 4.2 |  | 26.2 | -27.2 |  | 28.8 | 0.2 |  |
|  | *Bin. var* | | *100.0* |  |  | *100.0* |  |  | *100.0* |  |  |
|  | TD bin. var | | 13.6 | 278.8 |  | 9.5 | 495.2 |  | 44.2 | 380.8 |  |
|  | A*stage | | 33.3 | 17.4 |  | 52.4 | 3.1 |  | 67.3 | -4.9 |  |
|  | TD A*stage | | 3.0 | 89.4 |  | 0.0 |  |  | 19.2 | -15.7 |  |
|  | A*dep | | 19.7 | 23.1 |  | 7.1 | 19.0 |  | 15.4 | -27.4 |  |
|  | TD A*dep | | 3.0 | -15.8 |  | 0.0 |  |  | 5.8 | -58.8 |  |
|  | A*bin. var | | 54.5 | 278.8 |  | 28.6 | 495.2 |  | 55.8 | 380.8 |  |
|  | TD A*bin. var | | 1.5 | 278.8 |  | 0.0 |  |  | 17.3 | 380.8 |  |
| **Df - TD Cscore included** | | | |  |  |  |  |  |  |  |  |
|  | Stage | | 100.0 | 0.0 |  | 100.0 | 0.0 |  | 100.0 | 0.0 |  |
|  | TD stage | | 100.0 | 0.0 |  | 100.0 | 0.8 |  | 100.0 | 0.4 |  |
|  | Deprivation | | 100.0 | 0.0 |  | 100.0 | 0.0 |  | 100.0 | 0.0 |  |
|  | TD dep | | 22.2 | 122.2 |  | 25.0 | 316.7 |  | 13.0 | -29.1 |  |
|  | Age | | 55.6 | -16.3 |  | 25.0 | -58.6 |  | 69.6 | -3.9 |  |
|  | NL Age | | 44.4 | 32.3 |  | 75.0 | 89.4 |  | 30.4 | 10.3 |  |
|  | TD age | | 55.6 | 15.7 |  | 25.0 | -30.6 |  | 34.8 | 20.8 |  |
|  | Bin. var | | 100.0 | 278.8 |  | 100.0 | 495.2 |  | 100.0 | 380.8 |  |
|  | TD bin. var | | 100.0 |  |  | 100.0 |  |  | 100.0 |  |  |
|  | A*stage | | 11.1 | -60.9 |  | 25.0 | -50.8 |  | 73.9 | 4.4 |  |
|  | TD A*stage | | 0.0 | -100.0 |  | 0.0 |  |  | 21.7 | -4.7 |  |
|  | A*dep | | 11.1 | -30.6 |  | 25.0 | 316.7 |  | 4.3 | -79.5 |  |
|  | TD A*dep | | 0.0 | -100.0 |  | 0.0 |  |  | 0.0 | -100.0 |  |
|  | A*bin. var | | 44.4 | 208.6 |  | 0.0 | -100.0 |  | 43.5 | 274.8 |  |
|  | TD A*bin. var | | 11.1 | 2677.8 |  | 0.0 |  |  | 39.1 | 987.0 |  |
| **Dg -A*dep included** | | |  |  |  |  |  |  |  |  |  |
|  | Stage | | 100.0 | 0.0 |  | 100.0 | 0.0 |  | 100.0 | 0.0 |  |
|  | TD stage | | 100.0 | 0.0 |  | 100.0 | 0.8 |  | 100.0 | 0.4 |  |
|  | Deprivation | | 100.0 | 0.0 |  | 100.0 | 0.0 |  | 100.0 | 0.0 |  |
|  | TD dep | | 37.5 | 275.0 |  | 6.7 | 11.1 |  | 67.9 | 269.2 |  |
|  | Age | | 70.0 | 5.4 |  | 53.3 | -11.7 |  | 73.6 | 1.6 |  |
|  | NL Age | | 30.0 | -10.7 |  | 46.7 | 17.8 |  | 26.4 | -4.3 |  |
|  | TD age | | 60.0 | 25.0 |  | 46.7 | 29.6 |  | 17.0 | -41.0 |  |
|  | Bin. var | | 32.5 | 23.1 |  | 20.0 | 19.0 |  | 15.1 | -27.4 |  |
|  | TD bin. var | | 2.5 | -30.6 |  | 6.7 | 316.7 |  | 1.9 | -79.5 |  |
|  | A*stage | | 27.5 | -3.2 |  | 46.7 | -8.1 |  | 77.4 | 9.3 |  |
|  | TD A*stage | | 5.0 | 212.5 |  | 0.0 |  |  | 11.3 | -50.3 |  |
|  | *A*dep* | | *100.0* |  |  | *100.0* |  |  | *100.0* |  |  |
|  | TD A*dep | | 22.5 | 525.0 |  | 0.0 |  |  | 66.0 | 371.7 |  |
|  | A*bin. var | | 22.5 | 56.3 |  | 6.7 | 38.9 |  | 7.5 | -34.9 |  |
|  | TD A*bin. var | | 0.0 | -100.0 |  | 0.0 |  |  | 0.0 | -100.0 |  |
| **Dh - A*stage missed** | | |  |  |  |  |  |  |  |  |  |
|  | Stage | | 100.0 | 0.0 |  | 100.0 | 0.0 |  | 100.0 | 0.0 |  |
|  | TD stage | | 100.0 | 0.0 |  | 98.4 | -0.8 |  | 98.6 | -1.0 |  |
|  | Deprivation | | 100.0 | 0.0 |  | 100.0 | 0.0 |  | 100.0 | 0.0 |  |
|  | TD dep | | 11.2 | 11.7 |  | 6.5 | 8.4 |  | 13.7 | -25.6 |  |
|  | Age | | 59.2 | -10.8 |  | 48.8 | -19.2 |  | 63.0 | -13.0 |  |
|  | NL Age | | 40.8 | 21.4 |  | 51.2 | 29.3 |  | 37.0 | 34.0 |  |
|  | TD age | | 39.1 | -18.5 |  | 29.3 | -18.7 |  | 26.0 | -9.6 |  |
|  | Bin. var | | 24.6 | -6.9 |  | 16.3 | -3.2 |  | 23.3 | 12.0 |  |
|  | TD bin. var | | 4.5 | 24.1 |  | 2.4 | 52.4 |  | 8.2 | -10.7 |  |
|  | *A*stage* | | *0.0* |  |  | *0.0* |  |  | *0.0* |  |  |
|  | TD A*stage | | 0.0 | -100.0 |  | 0.0 |  |  | 0.0 | -100.0 |  |
|  | A*dep | | 16.2 | 1.3 |  | 6.5 | 8.4 |  | 16.4 | -22.5 |  |
|  | TD A*dep | | 3.9 | 8.6 |  | 0.0 |  |  | 8.2 | -41.3 |  |
|  | A*bin. var | | 11.7 | -18.5 |  | 0.0 | -100.0 |  | 12.3 | 6.3 |  |
|  | TD A*bin. var | | 0.6 | 39.7 |  | 0.0 |  |  | 2.7 | -23.9 |  |
