# Supplementary material for: Comparison of model-building strategies for excess hazard regression models in the context of cancer epidemiology
Source: BMC Med Res Methodol. 2019 Nov 20;19:210. doi: 10.1186/s12874-019-0830-9 (PMC6869178; doi:10.1186/s12874-019-0830-9)
Supplement: Supplementary file 5 — Additional file 5. Effects of age, generating (red), estimated (grey), averaged (black) for all models selected by each algorithm, scenario A-D. [file 12874_2019_830_MOESM5_ESM.docx]

**Additional file 5**

Effects of age, generating (red), estimated (grey), averaged (black) for all models selected by each algorithm, scenario A-D

**Impact of relaxing some of the features of the generating models (non-linearity, time dependency, interactions of age at diagnosis) on the hazard ratio for age, survival and cumulative hazard**

**HR for the 90th age percentile (age 85.3) versus the 10th age percentile (age 58.7) by scenario when adding assumptions to the simulation models - Full cohort of lung cancer patients**

| **B** |  |  |  |  |  |  |  |  |  |
| --- | --- | --- | --- | --- | --- | --- | --- | --- | --- |
|  |  | HR age* | | | | |  |  |  |
| Time | Stage | Scenario B | |  | No interaction | |  |  |  |
| All | All |  |  |  | 1.75 | |  |  |  |
|  |  |  |  |  | 1.67 | 1.83 |  |  |  |
| All | 1 | 2.48 | |  |  |  |  |  |  |
|  |  | 2.01 | 3.07 |  |  |  |  |  |  |
| All | 2 | 2.69 | |  |  |  |  |  |  |
|  |  | 2.20 | 3.28 |  |  |  |  |  |  |
| All | 3 | 1.68 | |  |  |  |  |  |  |
|  |  | 1.53 | 1.85 |  |  |  |  |  |  |
| All | 4 | 1.68 | |  |  |  |  |  |  |
|  |  | 1.59 | 1.78 |  |  |  |  |  |  |
| **C** |  |  |  |  |  |  |  |  |  |
|  |  | HR age* | | | | | | | |
| Time | Stage | Scenario C | |  | L TD age | |  | NL P age | |
| All (95% CI) |  |  |  |  |  |  |  | 1.80 | |
|  |  |  |  |  |  |  |  | 1.72 | 1.88 |
| 1m (95% CI) | All | 1.96 | |  | 1.95 | |  |  |  |
|  |  | 1.85 | 2.08 |  | 1.84 | 2.07 |  |  |  |
| 6m (95% CI) | All | 1.71 | |  | 1.65 | |  |  | |
|  |  | 1.63 | 1.80 |  | 1.57 | 1.73 |  |  |  |
| 12m (95% CI) | All | 1.63 | |  | 1.55 | |  |  | |
|  |  | 1.52 | 1.73 |  | 1.46 | 1.65 |  |  |  |

| **D** |  |  |  |  |  |  |  |  |  |  |  |  |  |  |  |  |  |  |
| --- | --- | --- | --- | --- | --- | --- | --- | --- | --- | --- | --- | --- | --- | --- | --- | --- | --- | --- |
|  |  | HR age* | | | | | | | | | | | | | | | | |
| Time | Stage | Scenario D | |  | L TD age | |  | NL P age | |  | L P age | |  | No interaction (NL TD age) | |  | No interaction (LP age) | |
| All times (95% CI) | All |  |  |  |  |  |  |  |  |  |  |  |  |  |  |  | 1.75 | |
|  |  |  |  |  |  |  |  |  |  |  |  |  |  |  |  |  | 1.67 | 1.83 |
|  | 1 |  |  |  |  |  |  | 2.67 | |  | 2.66 | |  |  |  |  |  |  |
|  |  |  |  |  |  |  |  | 2.18 | 3.27 |  | 2.15 | 3.30 |  |  |  |  |  |  |
|  | 2 |  |  |  |  |  |  | 3.01 | |  | 2.94 | |  |  |  |  |  |  |
|  |  |  |  |  |  |  |  | 2.50 | 3.63 |  | 2.40 | 3.60 |  |  |  |  |  |  |
|  | 3 |  |  |  |  |  |  | 1.85 | |  | 1.79 | |  |  |  |  |  |  |
|  |  |  |  |  |  |  |  | 1.69 | 2.03 |  | 1.63 | 1.97 |  |  |  |  |  |  |
|  | 4 |  |  |  |  |  |  | 1.66 | |  | 1.62 | |  |  |  |  |  |  |
|  |  |  |  |  |  |  |  | 1.57 | 1.76 |  | 1.54 | 1.71 |  |  |  |  |  |  |
| 1m(95% CI) | All |  |  |  |  |  |  |  |  |  |  |  |  | 1.96 | |  |  |  |
|  |  |  |  |  |  |  |  |  |  |  |  |  |  | 1.85 | 2.08 |  |  |  |
|  | 1 | 3.61 | |  | 3.71 | |  |  |  |  |  |  |  |  |  |  |  |  |
|  |  | 2.90 | 4.49 |  | 2.96 | 4.66 |  |  |  |  |  |  |  |  |  |  |  |  |
|  | 2 | 3.93 | |  | 3.97 | |  |  |  |  |  |  |  |  |  |  |  |  |
|  |  | 3.21 | 4.81 |  | 3.20 | 4.92 |  |  |  |  |  |  |  |  |  |  |  |  |
|  | 3 | 2.30 | |  | 2.30 | |  |  |  |  |  |  |  |  |  |  |  |  |
|  |  | 2.06 | 2.57 |  | 2.06 | 2.57 |  |  |  |  |  |  |  |  |  |  |  |  |
|  | 4 | 1.83 | |  | 1.84 | |  |  |  |  |  |  |  |  |  |  |  |  |
|  |  | 1.72 | 1.95 |  | N/A | N/A |  |  |  |  |  |  |  |  |  |  |  |  |
| 6m (95% CI) | All |  |  |  |  |  |  |  |  |  |  |  |  | 1.71 | |  |  |  |
|  |  |  |  |  |  |  |  |  |  |  |  |  |  | 1.63 | 1.80 |  |  |  |
|  | 1 | 2.86 | |  | 2.88 | |  |  |  |  |  |  |  |  |  |  |  |  |
|  |  | 2.33 | 3.51 |  | 2.33 | 3.56 |  |  |  |  |  |  |  |  |  |  |  |  |
|  | 2 | 3.12 | |  | 3.08 | |  |  |  |  |  |  |  |  |  |  |  |  |
|  |  | 2.58 | 3.77 |  | 2.51 | 3.77 |  |  |  |  |  |  |  |  |  |  |  |  |
|  | 3 | 1.83 | |  | 1.78 | |  |  |  |  |  |  |  |  |  |  |  |  |
|  |  | 1.66 | 2.00 |  | 1.62 | 1.96 |  |  |  |  |  |  |  |  |  |  |  |  |
|  | 4 | 1.46 | |  | 1.42 | |  |  |  |  |  |  |  |  |  |  |  |  |
|  |  | 1.36 | 1.56 |  | 1.34 | 1.51 |  |  |  |  |  |  |  |  |  |  |  |  |
| 12m (95% CI) | All |  |  |  |  |  |  |  |  |  |  |  |  | 1.63 | |  |  |  |
|  |  |  |  |  |  |  |  |  |  |  |  |  |  | 1.52 | 1.73 |  |  |  |
|  | 1 | 2.62 | |  | 2.61 | |  |  |  |  |  |  |  |  |  |  |  |  |
|  |  | 2.13 | 3.21 |  | 2.11 | 3.23 |  |  |  |  |  |  |  |  |  |  |  |  |
|  | 2 | 2.85 | |  | 2.79 | |  |  |  |  |  |  |  |  |  |  |  |  |
|  |  | 2.36 | 3.45 |  | 2.28 | 3.42 |  |  |  |  |  |  |  |  |  |  |  |  |
|  | 3 | 1.67 | |  | 1.61 | |  |  |  |  |  |  |  |  |  |  |  |  |
|  |  | 1.51 | 1.84 |  | 1.46 | 1.78 |  |  |  |  |  |  |  |  |  |  |  |  |
|  | 4 | 1.33 | |  | 1.29 | |  |  |  |  |  |  |  |  |  |  |  |  |
|  |  | 1.22 | 1.45 |  | 1.20 | 1.39 |  |  |  |  |  |  |  |  |  |  |  |  |
| * HR for age 85.3 (90th percentile) vs. 58.7 (10th percentile) | | | | | | | | | | | | | | | | | | |
