# Supplementary material for: Comparison of model-building strategies for excess hazard regression models in the context of cancer epidemiology
Source: BMC Med Res Methodol. 2019 Nov 20;19:210. doi: 10.1186/s12874-019-0830-9 (PMC6869178; doi:10.1186/s12874-019-0830-9)
Supplement: Supplementary file 7 — Additional file 7. Original (red line), estimated (grey lines - time varying, box-plot - time fixed) and averaged (black line) cohort net survival. Scenario A-D. [file 12874_2019_830_MOESM7_ESM.docx]

**Additional file 7**

Original (red line), estimated (grey lines - time varying, box-plot - time fixed) and averaged (black line) cohort net survival. Scenario A-D

Mean *pABCtime* values of the estimated cohort survival estimates obtained using the non-parametric Pohar Perme estimator

| **Cohort net survival** | *Stage at diagnosis* | **A** |  | **B** |  | **C** |  | **D** |
| --- | --- | --- | --- | --- | --- | --- | --- | --- |
| Pohar Perme estimator | I | 1.96% |  | 2.13% |  | 2.51% |  | 2.08% |
|  | II | 4.56% |  | 4.44% |  | 7.09% |  | 4.90% |
|  | III | 3.80% |  | 3.21% |  | 3.21% |  | 3.75% |
|  | IV | 6.11% |  | 4.70% |  | 8.07% |  | 6.00% |

Distribution of individual pABCtime values for the cohort survival estimates obtained non-parametrically or through model selection.
