# Supplementary material for: Comparison of model-building strategies for excess hazard regression models in the context of cancer epidemiology
Source: BMC Med Res Methodol. 2019 Nov 20;19:210. doi: 10.1186/s12874-019-0830-9 (PMC6869178; doi:10.1186/s12874-019-0830-9)
Supplement: Supplementary file 8 — Additional file 8. Integrated absolute estimated bias for the effects of age, stage, deprivation and the extra binary variable, by scenario (A-D) and for each model selection algorithm. [file 12874_2019_830_MOESM8_ESM.docx]

**Additional file 8**

Integrated absolute estimated bias for the effects of age, stage, deprivation and the extra binary variable, by scenario (A-D) and for each model selection algorithm

Scenario A

Scenario C

Scenario B

Scenario D
